# The effect of periodontal treatments on endothelial function in degrees of periodontitis patients: A systematic review and meta-analysis

**DOI:** 10.1371/journal.pone.0308793

**Published:** 2024-09-19

**Authors:** Jingzhe Lyu, Yiyao Zhang, Run Zhou, Cheng Ding, Hong Ye, Qian Fang, Chunhui Jiang, Xijie Chen, Liangjun Zhong

**Affiliations:** 1 Department of Periodontics, School of Stomatology, Hangzhou Normal University, Hangzhou, China; 2 Department of Stomatology, Affiliated Hospital of Hangzhou Normal University, Hangzhou, Zhejiang, P. R. China; 3 Melbourne Dental School, The University of Melbourne, Melbourne, Australia; 4 School of Nursing, Hangzhou Normal University, Hangzhou, China; 5 Stomatology Center, Affiliated Hospital of Hangzhou Normal University, Hangzhou, China; 6 School of Stomatology, Hangzhou Normal University, Hangzhou, China; 7 Department of Stomatology, Affiliated Hospital of Hangzhou Normal University, Hangzhou, Zhejiang, P. R. China; Federal University of Minas Gerais: Universidade Federal de Minas Gerais, BRAZIL

## Abstract

**Objective:**

This article focus on patients with moderate-to-severe periodontitis and periodontitis patients with cardiovascular disease. After they received periodontal initial therapy or antimicrobial drug treatment, was there any improvement in endothelial function during short- and long-term followups?

**Method:**

Relevant randomized controlled trials and clinical trials up to 30^th^ June 2024 were identified and retrieved from electronic databases including PubMed, Cochrane Library, Web of Science and CNKI databases, with periodontitis therapy, periodontal disease and endothelial function as the keywords. The weighted **(**WMD**)** or standardized mean difference (SMD) was calculated using a fixed- or random-effect model and assessed heterogeneous results.

**Result:**

Generally, 14 studies published between 2004 and 2022 were eligible for the meta-analysis, which are all randomised clinical trials. A total of 491 periodontitis patients were screened. All participants received whole-mouth supragingival and subgingival scaling and root planing of the teeth, some trials combined with antimicrobial drug treatment as well as extracting teeth that could not be saved. The outcome indicators were measured by flow-mediated dilatation(FMD**)** levels. The results of the short term (≤3 months) periodontitis initial therapy group showed positive results (WMD = -3.78,95%CI = [-5.49,-2.07], P<0.0001), while the results of the long term (6 months) periodontitis therapy group exhibited significant difference (WMD = -0.96,95%CI = [-2.06,0.14],P = 0.09). Furthermore, study population were categorized according to the severity of periodontitis, the presence of comorbidities, endothelial dysfunction, and the inclusion of extractions and antimicrobial therapy in the treatment process. The effects of each of these factors on FMD were explored and the results of these subgroups all support periodontitis therapy.

**Conclusion:**

The results showed that periodontal treatment enhances endothelial function. Additionally, after subgroup analysis of long-term and short-term follow-up, patients with severe periodontitis, and different periodontal treatments, periodontal therapy was shown to increase FMD levels.

## Introduction

Periodontitis is characterized by chronic and progressive bacterial infection of the gingiva leading to alveolar bone destruction and loss of soft tissue attachment to the teeth. Additionally, periodontal infection is not only harmful to oral health but is also correlated to a variety of systemic diseases. Epidemiological studies suggest a link between severe periodontitis and atherosclerosis [1,2]. Although the mechanisms accounting for such a relationship have not been fully defined, investigators have proposed that periodic transient bacteremia could lead to the invasion of vascular cells and increase the levels of circulating cytokines accelerating the atherogenic process [3].

Endothelial dysfunction (ED) is considered to be one of the major pathological mechanisms in which hypertension occurs at the early stages of atherogenesis. A normal endothelium ensures the appropriate stiffness and elasticity of the blood vessel walls, exerts an anti-inflammatory and anti-coagulant effect, and limits smooth muscle proliferation and leukocyte migration to the vessel walls [4]. A likely target for circulating cytokines and oral pathogens is the vascular endothelium, which plays a central role in the regulation of vascular homeostasis [5]. The circulating cytokines and oral pathogens prevalent in patients with periodontitis may also activate endothelial cells, increasing the genetic expression of adhesion molecules and suppressing the production of nitric oxide [6,7].

There have been a series of studies on the evaluation of vascular endothelial function. Currently, several invasive and noninvasive methods are commonly used to evaluate vascular function. Invasive tests require the injection of acetylcholine into human coronary arteries, but their clinical application is limited because they are invasive, time-consuming, and expensive. Furthermore, they are not recommended for healthy or asymptomatic patients due to their invasive nature [8]. On the other hand, a noninvasive endothelial function detection method which first appeared in the 1990s also is highly prevalent today. With the continuous development of basic and clinical research, the medical industry has recently paid increasing attention to examining endothelial function in cardiovascular diseases, and noninvasive vascular endothelial function detection has become a hot research topic [9]. At present, brachial artery flow-mediated dilatation (FMD) is the most popular method for evaluating vascular endothelial function [10]. It has advantages of little to no trauma, relatively low cost, simple operation procedure, decent repeatability, wide application and is generally more acceptable [10]. Therefore, FMD detection has recently been widely adopted for cardiovascular research in clinical trials.

A number of studies have shown significant improvement in endothelial function for periodontitis treatment. Orlandi M. and colleagues [11] demonstrated an association between increased c-IMT and impaired FMD/PD. Data from intervention studies suggest that periodontal treatment has a beneficial effect on FMD indicating a potential improvement in endothelial function. However, significant heterogeneity was shown in the comparison of FMD levels between periodontitis (I^2^ = 80.1%). Similarly, the heterogeneity regarding the effect of periodontal treatment on EDD was high (I^2^ = 78%). This was possibly due in part because participants differed in the severity of periodontitis and differences in periodontitis treatment. Therefore, the author suspects that patients with different degrees of periodontitis and different treatments should be graded and discussed in separate groups. A meta-analysis demonstrated that periodontitis treatment improves atherosclerosis by performing an analysis of inflammatory biomarkers (IL-6, IL-1β, sVCAM-1, sE-selectin, etc.) [12]. Additionally, several clinical trials have demonstrated that periodontitis treatment reduces the risk of cardiovascular disease and improves systemic inflammation levels [13–15]. However, previous studies have shown that FMD is the "golden indicator" for the evaluation and measurement of endothelial function [16–18], but the outcome metrics in the above studies did not directly correlate with improvements endothelial function.

In this article, different groups were analyzed according to the severity of periodontitis, the method of periodontal treatment, and the duration of follow-up in the study population. Using FMD as an outcome indicator, we further screened clinical trials to discuss the effect of periodontitis treatment on the improvement of endothelial function under different circumstances. We hope this study can provide more evidence to support the effect of periodontal treatment on endothelial function.

## Method

This systematic review was conducted in accordance with the guidelines of Transparent Reporting of Systematic Reviews and Meta-Analyses [PRISMA statement (Moher et al. 2020)] (S1 Table). The protocol has been pre-registered in PROSPERO under the registration number CRD42023413528.

This study adhered to the PICO principles: P (population), the population was diagnosed with periodontitis in patients without systemic complications except cardiovascular disease and hypertension. I (intervention), the intervention was periodontitis initial therapy, which includes control of supragingival plaque by dental calculus removal and personalized oral hygiene instruction, and full-mouth subgingival scaling and root planing (SRP) under local anesthesia. Patients received individual periodontal maintenance or return visits (professional plaque biofilm removal and intensive oral hygiene instruction) once a month, 3 months, or 6 months. C (comparison), our comparison was carried out before and after patients received periodontitis initial therapy. O (outcome), our outcome used an ultrasound detection of brachial artery flow-mediated-dilation (FMD).

### Search strategy

The study search included all articles published up to June 30^th^, 2024 in four separate databases: PubMed, Cochrane Library, Web of Science, and CNKI with no language restriction. The following search model was constructed using Boolean operators as well as medical subject headings (MeSH terms) and free text terms. The keywords selected include periodontitis, periodontitis therapy, periodontitis treatment, periodontitis nursing, endothelial function and dysfunction, endothelial vascular, flow-mediated dilation, cardiovascular disease, and hypertension. The detailed search strategies used in the databases are available in S2 Table.

### Eligibility criteria

The included studies had to fulfill the following criteria:

1. Human randomized clinical trials
2. Periodontitis patients without other systemic diseases (except cardiovascular diseases and hypertension)
3. Patients must receive periodontitis initial therapy
4. The measure of endothelial function was brachial artery flow-mediated vasodilator function,
5. Follow-up period of at least 1 month

The exclusion criteria were as follows:

1. Received periodontitis therapy in the past 6 months or was using antibiotics or anti-inflammatory drugs in the 3 months prior to the inclusion in the trial
2. Associated diseases other than cardiovascular disease or hypertension (including diabetes, cancer, etc.)
3. Other study designs, such as case series, cohort studies, or animal studies
4. Studies that included participants with conditions like pregnancy or breastfeeding

### Heterogeneity of trials

The heterogeneity across trials was detailed according to the following:

- Method of periodontal intervention
- Population characteristics
- Follow-up time for treatment
- Periodontitis severity

### Data extraction

Two authors (J.Z. Lyu and C. Ding) independently read the full texts of all potentially eligible articles. If there is disagreements, a third author (L.J.Zhong) is approached for discussion. Data were extracted from the full-text studies which met the inclusion criteria and recorded in a standardized data collection form. Disagreements were resolved through discussion between the authors to reach a consensus. For each study, the following essential information was documented: study location, funding source, study type, sample size, patients’ age, systemic disease, interventions, follow-up time, probing depth, clinical attachment loss, BOP, smoking status.

### Study quality assessment

The risk of bias was evaluated by the Cochrane Collaboration’s Risk-of-Bias 2 (ROB 2) tool, which is an update to the original risk of bias tool that launched in 2008. ROB 2 assesses the risk of bias in seven aspects on three grades: low risk, some concerns, and high risk. The 2019 revision of RoB2 sets out 5 evaluation domains: randomization process, deviation from intended interventions, missing outcome data, measurement of outcome and selection of the reported result. It focuses on controlling bias with appropriate statistical methods, which makes the evaluation more procedural and standardized, and also greatly reduces the interference of human factors. Finally, two authors (Zhang Y.Y., Ye H.) conducted risk assessments of the 14 included studies.

### Certainty of evidence

For studies included in the meta-analysis, Two reviewers, Run Zhou and Jingzhe Lyu used GRADEpro GDT software (GRADEpro GDT 2022) to determine the quality of evidence according to the Cochrane-recommended GRADE domains: risk of bias, inconsistency, indirectness, imprecision, and publication bias. If limitations were identified, the quality (certainty) of evidence was downgraded according to the guidelines.

### Data synthesis and meta-analysis

Statistical analyses were carried out using Review Manager 5.4.1 (Cochrane Collaboration, Oxford, UK). The FMD levels were presented as Means ± SD. The distribution of potential confounding variables (such as gender and age) that may influence the FMD levels was compared. Meta-analyses are displayed as forest plots. Continuous outcomes are calculated as weighted mean differences (WMDs) or standardized mean differences (SMDs). Statistical heterogeneity was estimated by Higgins’s I^2^ test I^2^ = 0, no heterogeneity, I^2^ ≤ 50%, low heterogeneity, I^2^ >50%, high heterogeneity [19]. If a statistically significant heterogeneity was found, a random-effect model was used; otherwise, a fixed-effect model was applied. A subgroup analysis was conducted based on the follow-up period (i.e. ≤3 months or 6 months), severity of Periodontitis, differences in initial treatments, and patients with comorbidities.

## Results

### Database search

**Fig 1:**
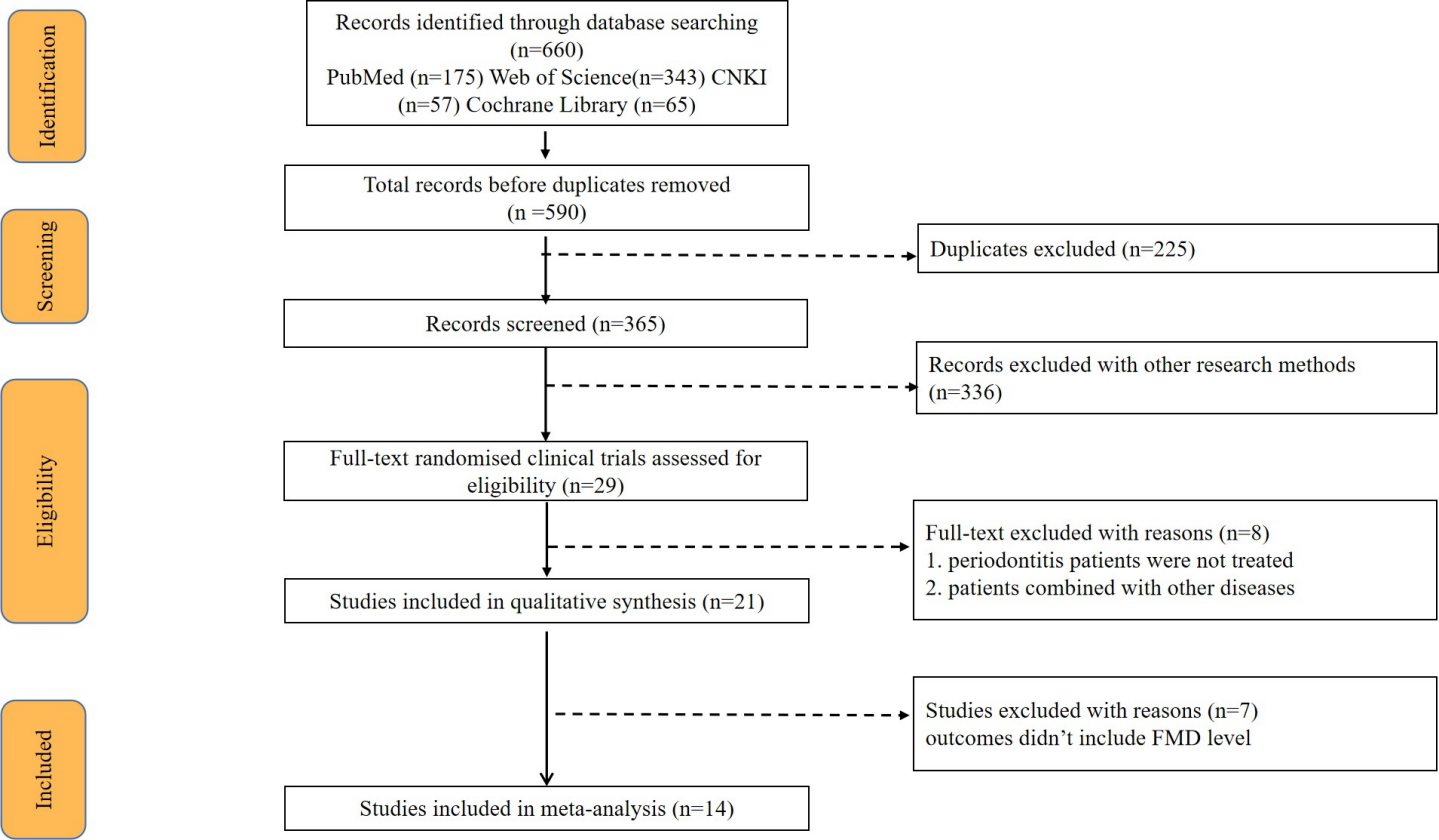
Flow diagram. A search was conducted in four databases, including Pubmed, Web of Science, CNKI and Cochrane. 14 articles were finally selected from 660 papers for meta-analysis.

The process used for selecting studies in the systematic review is outlined in Fig 1. In the initial search, 660 articles were found on PubMed, Web of Science, Cochrane Library, and the CNKI database. Before screening, 225 duplicates were eliminated. 336 Records were excluded because they did not meet the selection criteria for clinical trials. After reviewing 29 titles and abstracts, patients in 8 articles were excluded for not being treated or had a comorbidity with another disease. After further full-text analysis for the remaining 21 papers, 7 studies were excluded due to the lack of FMD data. All in all, 14 studies were retained in the Meta-analysis.

In total, there were 491 patients with periodontitis with all of them receiving periodontitis initial therapy. Before the therapy, all studies recorded periodontal clinical indicators and FMD levels as a baseline. 406 participants examined FMD levels again ≤ 3 months, while 155 patients examined them after 6 months. By comparing changes of FMD levels before and after treatment, we can determine the relationship between periodontitis initial treatment and FMD levels.

### Characteristics of the included studies

The characteristics of the included studies are shown in S3 Table. Details of the intervention, duration of follow-up, clinical periodontal indicators at baseline, and presence of smoking were included in the study characteristics. Most of the studies had a balanced gender distribution between the periodontitis and control groups, which had no effect on the experimental results.

### Study quality assessment

**Fig 2:**
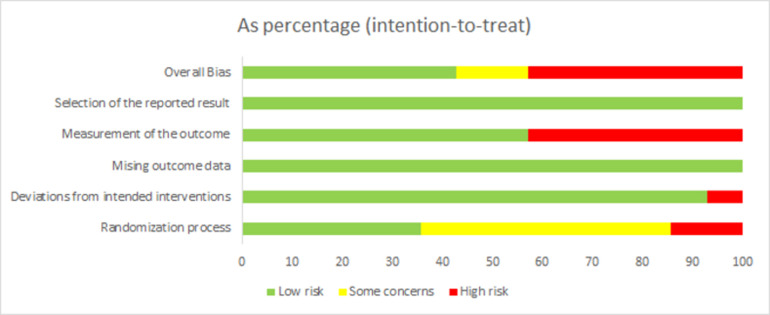
Risk of bias graph. This figure summarizes the risk of bias for all articles included in five sections.

**Fig 3:**
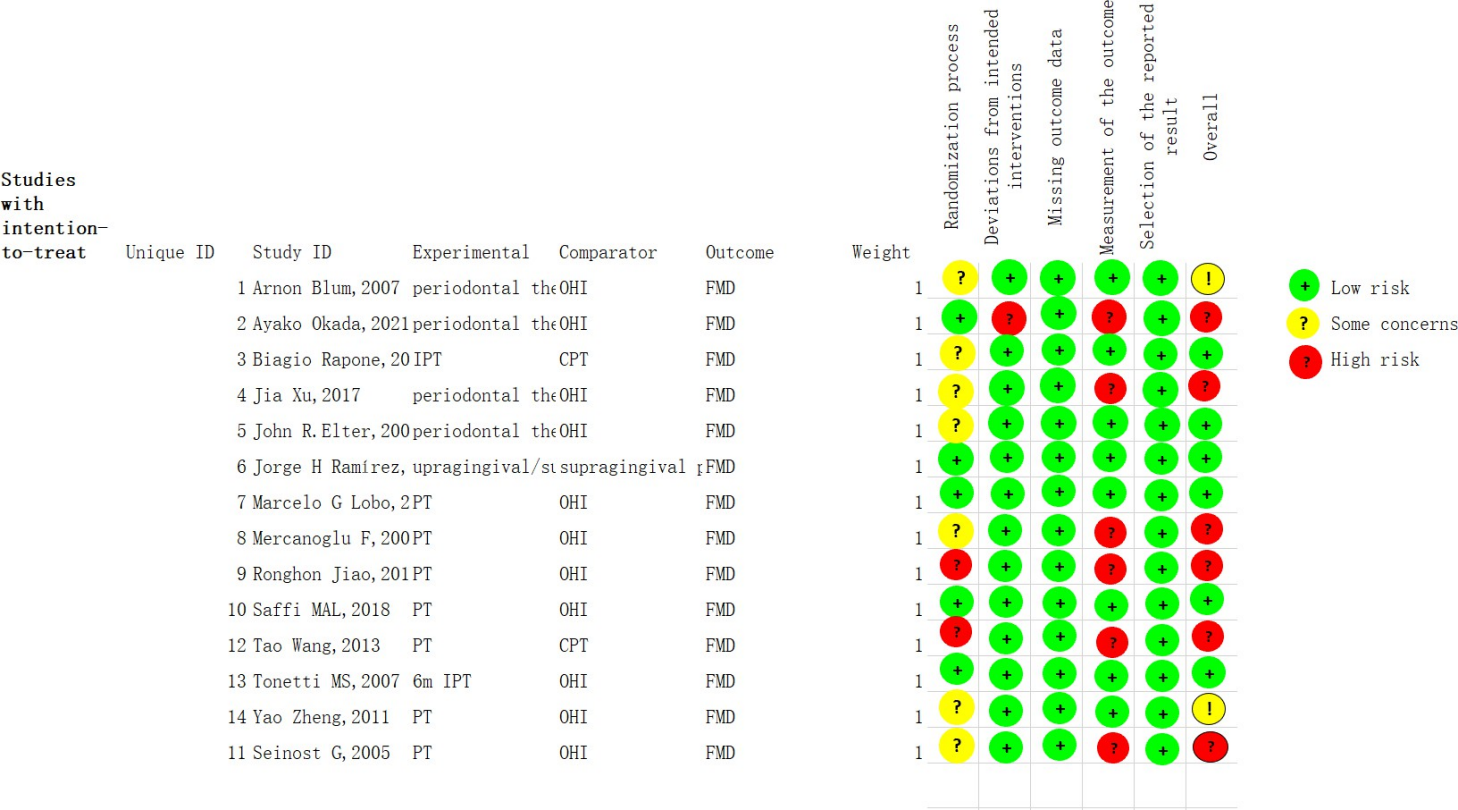
Risk of bias summary. This picture shows the bias analysis for each included article. All randomized controlled trials used the FMD level as outcome indicators, with intensive periodontal treatment in the experimental group and oral hygiene promotion or no treatment in the control group.

Among the 14 included studies, 6 had an overall high risk of bias and 2 study had “some concerns” on the overall risk of bias. The judgments for each domain of each risk of bias outcome are detailed in Figs 2 and 3. Risk of bias graph and summary Half of the studies did not explicitly state whether or not they were randomized during the random allocation process, 35.7% showed low risk, and 5 explicitly showed allocation sequence concealed in the process. The majority of trials had subjects and interveners who were aware of the intervention but there were not deviations from the intended intervention that arose because of the experimental context did. Additionally, they used an appropriate analysis to estimate the effect of assignment to intervention, so it showed a low risk of bias (92.9%) due to deviations from the intended interventions. The outcomes of all the trials were estimated sufficiently and led the low bias by missing outcomes data. Nevertheless, six studies picted that outcome assessors aware of the intervention received by study participants, potentially affecting the outcome metrics, with 42.9% at high risk and 57.1% at low risk.

### Grade assessment

All results of the verification certainty assessment can be found in S4 Table. To support the grade assessment, additional subgroup analyses were conducted to further explore the effects of periodontitis treatment on endothelial function. The reduction in study certainty mainly from the risk of bias, which was very high in five of the studies. 3 studies have been clinically registered, increasing the credibility of the studies. (The registration number is provided in the S5 Table). Inconsistency, indirectness, and imprecision were not serious in all studies, and some studies may have been influened by confounding factors. Overall, all but one of the studies were highly certainty.

### Meta-analysis results

Based on follow-up time, we performed a summary analysis of the 14 studies. A total of 491 participants showed a significant increase in FMD levels after periodontal treatment and a significant difference compared to baseline (Fig 4.1 in S1 Appendix WMD = -3.5-, 95%CI = [-4.96,-2.05],P<0.00001, I^2^ = 93%). Due to the high degree of heterogeneity in the results, we categorized them according to follow-up time. 9 studies followed patients for 3 months and 1 study followed patients for 1 month (John R. Elter). One study followed up for 6 weeks (Fehmi Mercanoglu), one study followed up for 3 and 6 months (Biagio Rapone), and the remaining two studies followed up for 6 months. Therefore, we first compared the effects of short-term (≤3 months) versus long-term (6 months) periodontitis follow-up treatment on endothelial function.

Ultimately, 406 periodontitis patients were selected in a meta-analysis of short-term follow-up (Fig 5.1 in S1 Appendix), which showed a significant improvement in FMD levels after treatment compared to base line (WMD = -3.78,95%CI = [-5.49,-2.07], P<0.0001). However, there was one study that showed no improvement in FMD levels with periodontal treatment(Ayako, Okada), we found that in this study the control group patients use a custom-made prescription tray as an extra intervention in addition to standard self-care. The prescription tray included the use of hypochlorous acid water and electrolyzed disinfectant for dental use, which is different from control group treatment in other studies. This is possibly a contributing factor for the differing result. In addition, this study was designed as open-label trial, and high risk in both deviations from intended and measurement of outcome, resulting in overall high risk of bias. We used a random effects model and showed extremely high heterogeneity I^2^ (94%). Although the sample size was large, the funnel plot (Fig 5.2 in S1 Appendix) showed significant asymmetry and possible publication bias. Next, we analyzed the sources of sensitivity and the characteristics of the study (S3 Table) which showed that 343 patients were diagnosed with chronic severe periodontitis while 63 patients were diagnosed with chronic periodontitis. In addition to this, 38 patients had concomitant cardiovascular disease (Saffi MAL). Thus, the severity of periodontitis, as well as concomitant comorbidities of the research population may be sources of heterogeneity. In addition, due to uneven age distribution, which ranged from 18 to 70 years, may also be an important source of selection bias.

During the long-term follow-up, we screened 3 studies (Tonetti MS, Marcelo G. Lobo, Biagio Rapone), and the results of the meta-analysis (WMD = -0.96, 95% CI = [-2.06,0.14], P = 0.09) also showed support for periodontitis treatment, and showed high heterogeneity I^2^ = 59%, using a random effects model (Figs 6.1 and 6.2 in S1 Appendix). All results were positive after our sensitivity analysis adopting the one-by-one elimination method, indicating that the data was robust. 24 patients with periodontitis combined with ST-segment elevation myocardial infarction (Marcelo G. Lobo) did not show endothelial dysfunction in baseline FMD levels. All three studies showed an increase in FMD levels after 6 months. However, due to the small number of studies in this condition and the insufficient sample size, further clinical trials are still needed to clarify this further.

Due to the high heterogeneity of the results in the meta-analysis, we conducted the following subgroup analyses based on the inclusion criteria and interventions. 8 studies included in meta-analysis were of patients with severe periodontitis, a total of 343 (56.7%), which were analyzed using a random effects model Figs 7.1 and 7.2 in S1 Appendix, WMD = -2.76,95% CI = [-4.43,-1.09],P = 0.001, which was a positive result, but had a non-significant reduction in heterogeneity. Two studies included patients with cardiovascular disease, a total of 62 (10.2%), and the results of the fixed-effects model analysis were WMD = -2.20,95% CI = [-4.08,-0.31],P = 0.02,I^2^ = 0 (Figs 8.1 and 8.2 in S1 Appendix)

It has been shown in a relevant study [20] that the normal value of FMD is ≥6%. When the result is <6% it indicates the presence of vascular endothelial dysfunction. Among the included literatures, 6 studies showed endothelial dysfunction at baseline, so we analyzed the endothelial dysfunction group Figs 9.1 and 9.2 in S1 Appendix. The result was still positive with WMD = -5.16,95%CI = [-8.62,-1.70], P = 0.003, I^2^ = 94%. It is worth noting that after periodontitis treatment, FMD levels increased to the normal range in all except 1 study (AO). Consistent with the results of previous analyses, there were confounders in AO’s study that caused bias in the results, and the quality of evidence results were moderate (S4 Table).

In addition, there were four articles that were registered as clinical trials, and all of them were labeled with the registration number in the articles, which is important evidence regarding the reliability of the results for the clinical trial data. These had a lower risk of bias and higher quality (S5 Table), so these 4 articles were also subgroup analyzed shown Figs 10.1 an d10.2 in S1 Appendix. The results showed moderate heterogeneity I^2^ = 51%,WMD = -0.47,95%CI = [-1.29,0.35],P = 0.26.

Finally, We found that the experimental group interventions differed across studies so we analyzed the interventions of different studies. There were 5 studies with antibiotic medication combined in the treatment, and 2 studies with extraction of affected teeth with no retention value in the treatment. Other studies used only supragingival and subgingival cleaning treatments. Some findings indicate that treatment with azithromycin has a favorable effect on endothelial function in patients with documented coronary artery disease [21]. In addition, a case-contral study showed that after tooth extraction, biomarkers of systemic inflammation, vascular function, and metabolism (high sensitivity C-reactive protein, lipids, fibrinogen, oxidative stress, and endothelial function analyses) indicated significant variability [22]. Since both medication and extraction influence the level of FMD, these two cases were analyzed in subgroups separately. In the subgroup treated with antimicrobial medication Figs 11.1 and 11.2 in S1 Appendix, the results of the random-effects model were analyzed as follows (WMD = -3.73,95%CI = [-5.98–1.47], P = 0.001), the heterogeneity decreased to 84%. In the subgroup of extraction of affected teeth with no retention value Figs 12.1 and 12.2 in S1 Appendix (WMD = -1.31,95%CI = [-2.28,-0.35], P = 0.08, I^2^ = 0%). Subgroup analysis results still support periodontal treatment with reduced heterogeneity.

## Discussion

The results of this study suggest that basic periodontitis treatment increases levels of FMD and improves endothelial function. In previous studies, there are investigations regarding the relationship between periodontitis and FMD or endothelial function. By evaluating patients with advanced periodontitis, Amar S. and colleagues [23] found that advanced periodontal disease exhibited endothelial dysfunction and evidence of systemic inflammation, possibly placing them at increased risk for cardiovascular disease. Higashi Y. and colleagues [24] evaluated endothelial function in patients with both periodontitis and coronary artery disease (CAD) and concluded that periodontitis was a risk factor for endothelial dysfunction in patients with coronary artery disease. Furthermore, periodontitis is associated with endothelial dysfunction in patients with CAD through a decrease in nitric oxide bioavailability. Systemic inflammation may be, at least in part, a cause and predictor for progression of endothelial dysfunction.

Periodontitis is a risk factor for endothelial dysfunction and can exacerbate the inflammatory response and increase the risk of coronary heart disease. If coronary artery disease was combined with periodontitis, the inflammatory response will be more severe and endothelial dysfunction will be more severely impaired. However, if patients with periodontitis were provided with timely basic periodontitis treatment, it could not only reduce the accumulation of plaque in the patient’s oral cavity and reduce the occurrence of bacteremia, but also could reduce systemic inflammation as well as reduce the risk of coronary heart disease attacks. There is growing evidence that periodontal treatment can reduce systemic inflammation and improves endothelial function. Francesco D’Aiuto and colleagues [25] explored acute effects of periodontitis therapy on bio-markers of vascular health. Nicole Pischon and colleagues [26] explored the influence of periodontitis therapy on the regulation of soluble cell adhesion molecule expression in aggressive periodontitis patients. Fábio Vidal and colleagues [27] found that periodontitis therapy reduces plasma levels of Interleukin-6, C-Reactive Protein, and fibrinogen in patients with severe periodontitis and refractory arterial hypertension. However, the above studies only investigated the effect on inflammatory factors after periodontitis treatment and did not scientifically assess vascular endothelial function. On the other hand, FMD, an important measure of endothelial function, was also included in the study. And it has been demonstrated that periodontal treatment can increase FMD levels and improve endothelial function.

This study builds on previous studies to further analyze in depth the effects of different periodontal treatments on FMD levels in patients with varying degrees of periodontitis. Since there was a high degree of heterogeneity in the previous studies, we carefully grouped the study participants, treatments, and follow-up times. During a careful reading of the included researches, we found that patients did not have the same degree of periodontitis, for example, some patients with severe periodontitis also had cardiovascular disease. In addition, as for the treatment, the experimental group of some studies chose basic periodontal treatment combined with antibiotic therapy, surgical treatment, and extraction of nonfunctional teeth, while the control group chose to perform supragingival cleaning and personalized tray care. Moreover, there was also a difference in the follow-up time between short-term <3 months and long-term ≥3 months. All three of these factors contributed to the final meta-analysis results as a source of heterogeneity. After this article analyze the data in subgroups, the final results were shown to be in favor of periodontal therapy, which reinforces the improved effect of periodontal therapy on endothelial function. That’s the point and advantage of this article.

The clinical significance of this study is to further investigate whether appropriate periodontal treatment will improve both endothelial dysfunction and vascular inflammation, based on the identification of periodontal teeth as triggers of these two.After analysis, we found that endothelial function could be significantly improved after 3 months of conservative treatment. After 6 months of adherence to follow-up treatment, the periodontal treatment effect is cumulative and endothelial function is improved. Furthermore, in patients with severe periodontitis, treatment is not limited to supragingival and subgingival scaling, but the use of antibiotic therapy and extraction of non-reserved teeth can also significantly improve FMD levels. There is no doubt that this result is meaningful. Timely periodontal treatment plays an important role in reducing the risk of cardiovascular disease and preventing coronary artery disease.

At the same time, this research also has some limitations. We only analyzed one outcome index, FMD, however the topic of endothelial health is a complicated and deep topic that has many contributing factors. The improvement of endothelial function is not only determined by FMD, but also correlated with vascular biomarkers and plasma inflammatory factors. Therefore, statistical analysis of inflammatory factors should be included to improve the results. Second, fewer studies included in this meta-analysis have long-term follow-up of more than 6 months, and more clinical trials and follow-ups are needed to verify the impact of periodontitis therapy on endothelial function in the long-term. Third, in the process of bias analysis and quality of evidence analysis of the included articles, we found that due to the interference of confounding factors, such as the absence of double-blind, hidden experiments, which led to the poor quality of some of the literature. So screening for high quality randomized controlled trials is especially critical for meta-analysis.

In future studies, we suggest that investigators should conduct comprehensive rating analysis of outcome indicators to provide more evidence for the assessment of endothelial function. Meanwhile, the inclusion criteria of subjects should be strictly screened to control confounding factors and reduce selectivity bias and follow-up bias. Diversified subgroup analyses should be performed to reduce heterogeneity, improve the authenticity of the results, and provide more valuable reference standards for the clinic.

## Conclusions

Our findings have shown that periodontal treatment can be effective in enhancing endothelial function. However, the heterogeneity of the study results was high. We then performed subgroup analyses based on follow-up time, periodontitis severity, and treatment method, all of which supported periodontal treatment and reduced the heterogeneity of the study to some extent. Furthermore, more extensive and high-quality studies are needed to fully understand the cumulative effects of periodontitis treatment on endothelial function, as well as the effects on cardiovascular markers and inflammatory factors.

## Supporting information

S1 Table Prisma checklist. (DOCX)

S2 Table Search strategy. (DOCX)

S3 Table Characteristics of the included studies. (PDF)

S4 Table GRADE evidence profile. (PDF)

S5 Table Experimental registration status. (DOCX)

S6 Table Periodontitis criteria. (PDF)

S1 Appendix Figures of study results. (PDF)

S2 Appendix Supporting references. (DOCX)

S1 Dataset Minimal data set. (PDF)
