# Supplementary material for: The effect of periodontal treatments on endothelial function in degrees of periodontitis patients: A systematic review and meta-analysis
Source: PLoS One. 2024 Sep 19;19(9):e0308793. doi: 10.1371/journal.pone.0308793 (PMC11412498; doi:10.1371/journal.pone.0308793)
Supplement: S1 Table — (DOCX) [file pone.0308793.s001.docx]

| **Section and Topic** | **Item #** | **Checklist item** | **Location where item is reported** |
| --- | --- | --- | --- |
| **TITLE** | | |  |
| Title | 1 | The effect of periodontal treatments on endothelial function in degrees of periodontitis patients：a systematic review and meta-analysis | P1 |
| **ABSTRACT** | | |  |
| Abstract | 2 | This article focus on patients with moderate-to-severe periodontitis and periodontitis patients with cardiovascular disease. After they received periodontal initial therapy or antimicrobial drug treatment, was there any improvement in endothelial function during short- and long-term followups. | P2 |
| **INTRODUCTION** | | |  |
| Rationale | 3 | Many researchers concluded that periodontitis initial therapy of periodontitis can improve endothelial function. However, differences in screening conditions for patients and in treatment measures in these studies led to high heterogeneity. Therefore, we refined the previous studies by performing detailed subgroup categorization based on patient characteristics and treatments. | P2 |
| Objectives | 4 | We hope this study can provide more evidence to support the effect of periodontal treatment on endothelial function. Furthermore, We performed a subgroup analysis of heterogeneity in previous studies,and study population were categorized according to the severity of periodontitis, the presence of comorbidities, endothelial dysfunction, and the inclusion of extractions and antimicrobial therapy in the treatment process. | P2 |
| **METHODS** | | |  |
| Eligibility criteria | 5 | 1. Human randomized clinical trials 2. Periodontitis patients without other systemic diseases (except cardiovascular diseases and hypertension) 3. Patients must receive periodontitis initial therapy 4. The measure of endothelial function was brachial artery flow-mediated vasodilator function, 5. Follow-up period of at least 1 month | P4 |
| Information sources | 6 | The study search included all articles published up to 31th March 2024 in four databases: PubMed、Cochrane Library、Web of Science and CNKI databases with no language restriction. | P4 |
| Search strategy | 7 | The study search included all articles published up to March 31th, 2024 in four separate databases: PubMed, Cochrane Library, Web of Science, and CNKI with no language restriction. The following search model was constructed using Boolean operators as well as medical subject headings (MeSH terms) and free text terms. The keywords selected include periodontitis, periodontitis therapy, periodontitis treatment, periodontitis nursing, endothelial function and dysfunction, endothelial vascular, flow-mediated dilation, cardiovascular disease, and hypertension. In addition, a manual search for the original published text and references cited in the review article was performed. | P4 |
| Selection process | 8 | Two authors (J.Z. Lyu and C. Ding) independently read the full texts of all potentially eligible articles. If there is disagreements, a third author (L.J.Zhong) is approached for discussion. Data were extracted from the full-text studies which met the inclusion criteria and recorded in a standardized data collection form. Disagreements were resolved through discussion between the authors to reach a consensus. | P4-p5 |
| Data collection process | 9 | Two authors (J.Z. Lyu and C. Ding) independently read the full texts of all potentially eligible articles. If there is disagreements, a third author (L.J.Zhong) is approached for discussion. Data were extracted from the full-text studies which met the inclusion criteria and recorded in a standardized data collection form. Disagreements were resolved through discussion between the authors to reach a consensus. For each study, the following essential information was documented: study location, funding source, study type, sample size, patients’ age, systemic disease, interventions, follow-up time, probing depth, clinical attachment loss, BOP, smoking status. | P4-P5 |
| Data items | 10 | For each study, the following essential information was documented: study location, funding source, study type, sample size, patients’ age, systemic disease, interventions, follow-up time, probing depth, clinical attachment loss, BOP, smoking status. | P5 |
| Study risk of bias assessment | 11 | The Review Manager (RevMan) software (Cochrane Collaboration) was used to create the ROB graph and summary. | P6 |
| Effect measures | 12 | The FMD levels were presented as Means ± SD. The distribution of potential confounding variables (such as gender and age) that may influence the FMD levels was compared. Meta-analyses are displayed as forest plots. Continuous outcomes are calculated as weighted mean differences (WMDs) or standardized mean differences (SMDs). | P5 |
| Synthesis methods | 13a | Making the study intervention characteristics table and comparing against the planned groups for each synthesis. | P5 |
|  | 13b | We sent emails to contact the authors of the articles to obtain the missing data, and if the authors were not contacted, the study was not included in the statistics. | P5 |
|  | 13d | Statistical analyses were carried out using Review Manager 5.4.1 (Cochrane Collaboration, Oxford, UK). Meta-analyses are displayed as forest plots. Continuous outcomes are calculated as weighted mean differences (WMDs) or standardized mean differences (SMDs). | P6 |
|  | 13e | Statistical heterogeneity was estimated by Higgins’s I^2^ test I^2^ = 0, no heterogeneity, I^2^ ≤ 50%, low heterogeneity, I^2^ ＞50%, high heterogeneity. If a statistically significant heterogeneity was found, a random-effect model was used; otherwise, a fixed-effect model was applied. A subgroup analysis was conducted based on the follow-up period (i.e. ≤3 months or 6 months), severity of Periodontitis, differences in initial treatments, and patients with comorbidities. Publication bias was assessed by Egger’s tests. | P5 |
|  | 13f | The Grading of Recommendations Assessment, Development, and Evaluation (GRADE) items as proposed by the GRADE working group (Guyatt et al. 2008) was used for grading evidence included in this review. Two reviewers, Yiyao Zhang and Hong Ye, rated the quality of the evidence and strength of recommendations on the following aspects: risk of bias for the individual trials, consistency and precision among the study outcomes, directness of the study results, and the detection of publication bias. Any disagreements between the two reviewers was resolved after additional discussion. | P5 |
| Reporting bias assessment | 14 | Revised Cochrane Risk-of-bias Tool for Randomized Trials (RoB 2), | P6 |
| Certainty assessment | 15 | The Grading of Recommendations Assessment, Development, and Evaluation (GRADE) items as proposed by the GRADE working group (Guyatt et al. 2008) was used for grading evidence included in this review. | P5 |
| **RESULTS** | | |  |
| Study selection | 16a | The process used for selecting studies in the systematic review is outlined in Fig. 1. In the initial search, 581 articles were found on PubMed, Web of Science, Cochrane Library, and the CNKI database. Additional records were also identified through manual search (n=9). | P5 |
|  | 16b | Before screening, 225 duplicates were eliminated. 336 Records were excluded because they did not meet the selection criteria for clinical trials. After reviewing 29 titles and abstracts, patients in 8 articles were excluded for not being treated or had a comorbidity with another disease. After further full-text analysis for the remaining 21 papers, 7 studies were excluded due to the lack of FMD data.. All in all, 14 studies were retained in the Meta-analysis. | P5 |
| Study characteristics | 17 | The characteristics of the included studies are shown in appendix Table 1. | P5 |
| Risk of bias in studies | 18 | According to Revised Cochrane Risk-of-bias Tool for Randomized Trials (RoB 2), the overall quality of evidence at the outcome level was assessed and displayed in Fig. 2. | P6 |
| Results of individual studies | 19 | We first compared the effects of short-term (≤3 months) versus long-term (6 months) periodontitis follow-up treatment on endothelial function, which showed a significant improvement in FMD levels after treatment compared to base line（WMD=-3.94，95%CI= [-5.68,-2.21], P<0.00001).  Next, we analyzed the sources of sensitivity and the characteristics of the study (Appendix 1) which showed that 343 patients were diagnosed with chronic severe periodontitis while 63 patients were diagnosed with chronic periodontitis. In addition to this, 31 patients had concomitant cardiovascular disease (Saffi MAL). Thus, the severity of periodontitis, as well as concomitant comorbidities of the research population may be sources of heterogeneity. In addition, age differences may also be an important source of bias.  8 studies included in meta-analysis were of patients with severe periodontitis, a total of 343 (56.7%), which were analyzed using a random effects model Fig. 5.1, WMD=-2.76,95% CI=[-4.43,-1.09],P=0.001, which was a positive result, but had a non-significant reduction in heterogeneity. Two studies included patients with cardiovascular disease, a total of 62 (10.2%), and the results of the fixed-effects model analysis were WMD=-2.20,95% CI=[-4.08,-0.31],P=0.02,I2=0 (Fig. 5.2)  Finally, we analyzed the interventions of different studies, there were 5 studies with antibiotic medication combined in the treatment, and 2 studies with extraction of affected teeth with no retention value in the treatment. Since both medication and extraction influence the level of FMD, these two cases were analyzed in subgroups separately. In the subgroup treated with antimicrobial medication Fig. 5.5, the results of the random-effects model were analyzed as follows (WMD=-4.01,95%CI=[-6.52,-1.49], P=0.002), the heterogeneity decreased to 83%. In the subgroup of extraction of affected teeth with no retention value Fig. 5.6, (WMD=-1.46,95%CI=[-2.71,-0.21], P=0.02, I2=0%) | P6-P7 |
| Results of syntheses | 20a | Study population were categorized according to the severity of periodontitis, the presence of comorbidities, endothelial dysfunction, and the inclusion of extractions and antimicrobial therapy in the treatment process. | P6-P7 |
|  | 20b | The outcome indicators were measured by flow-mediated dilatation(FMD) levels. The results of the short term (≤3 months) periodontitis initial therapy group showed positive results（WMD=-3.94，95%CI= [-5.68,-2.21], P<0.001), while the results of the long term (6 months) periodontitis therapy group exhibited significant difference (WMD= -0.40，95%CI=[-0.59,-0.20],P＜0.001). | P6-P7 |
|  | 20c | The severity of periodontitis, the presence of comorbidities, endothelial dysfunction, and the inclusion of extractions and antimicrobial therapy in the treatment process. | P6-P7 |
|  | 20d | During the long-term follow-up, we screened 3 studies (Tonetti MS, Marcelo G. Lobo, Biagio Rapone), and the results of the meta-analysis (WMD=-0.40, 95% CI=[-0.59,-0.20], P<0.0001) also showed support for periodontitis treatment, and showed moderate heterogeneity I2=52%, using a fixed-effects model (Fig. 4). All results were positive after our sensitivity analysis adopting the one-by-one elimination method, indicating that the data was robust.  8 studies included in meta-analysis were of patients with severe periodontitis, a total of 343 (56.7%), which were analyzed using a random effects model Fig. 5.1, WMD=-2.76,95% CI=[-4.43,-1.09],P=0.001, which was a positive result, but had a non-significant reduction in heterogeneity. Two studies included patients with cardiovascular disease, a total of 62 (10.2%), and the results of the fixed-effects model analysis were WMD=-2.20,95% CI=[-4.08,-0.31],P=0.02,I2=0 (Fig. 5.2)  Among the included literatures, 6 studies showed endothelial dysfunction at baseline, so we analyzed the endothelial dysfunction group Fig. 5.3. The result was still positive with WMD=-6.54,95%CI=[-7.61,-5.47], P<0.00001, and the heterogeneity I2=29% was low and clinically significant. It is worth noting that after periodontitis treatment, FMD levels increased to the normal range in all except 1 study (AO). | P6-P7 |
| Reporting biases | 21 | According to Revised Cochrane Risk-of-bias Tool for Randomized Trials (RoB 2), the overall quality of evidence at the outcome level was assessed and displayed in Fig. 2. Furthermore, two authors (Zhang Y.Y., Ye H.) conducted risk assessments of the 14 included studies. | P6 |
| P5Certainty of evidence | 22 | The Grading of Recommendations Assessment, Development, and Evaluation (GRADE) items as proposed by the GRADE working group (Guyatt et al. 2008) was used for grading evidence included in this review. | P5 |
| **DISCUSSION** | | |  |
| Discussion | 23a | The results of this study suggest that basic periodontitis treatment increases levels of FMD and improves endothelial function.Furthermore, study population were categorized according to the severity of periodontitis, the presence of comorbidities, endothelial dysfunction, and the inclusion of extractions and antimicrobial therapy in the treatment process. The effects of each of these factors on FMD were explored and the results of these subgroups all support periodontitis therapy. | P7 |
|  | 23b | We only analyzed one outcome index, FMD, however the topic of endothelial health is a complicated and deep topic that has many contributing factors. The improvement of endothelial function is not only determined by FMD, but also correlated with vascular biomarkers and plasma inflammatory factors. Therefore, statistical analysis of inflammatory factors should be included to improve the results. | P8 |
|  | 23c | Fewer studies included in this meta-analysis have long-term follow-up of more than 6 months, and more clinical trials and follow-ups are needed to verify the impact of periodontitis therapy on endothelial function in the long-term. | P8 |
|  | 23d | The significance of this study is that timely periodontitis treatment can reduce the risk of cardiovascular disease and prevent endothelial dysfunction. | P8 |
| **OTHER INFORMATION** | | |  |
| Registration and protocol | 24 | The protocol has been pre-registered in PROSPERO under the registration number CRD42023413528. | P4 |
| Support | 25 | We gratefully acknowledge National Natural Science Foundation of China (NSFC) (22278103, 21  [672048](https://www.sciencedirect.com/science/article/pii/S0300571223000647?via=ihub#gs0001) and [81570989](https://www.sciencedirect.com/science/article/pii/S0300571223000647?via=ihub#gs0001)), Natural Science Foundation of Zhejiang Province (ZJNSF) ([LY19B020006](https://www.sciencedirect.com/science/article/pii/S0300571223000647?via=ihub#gs0002) and [LY15B020008](https://www.sciencedirect.com/science/article/pii/S0300571223000647?via=ihub#gs0002)), Hangzhou Biomedicine and Health Industry Development Support Technology Plan (2021WJCY050). Major Project of Hangzhou Health Science and Technology Plan ([Z20200046](https://www.sciencedirect.com/science/article/pii/S0300571223000647?via=ihub#gs0003)), and Key Subject of Stomatology in Hangzhou for financial support. | P8-P9 |
| Competing interests | 26 | The authors declare no conflict of interest. | P9 |
| Availability of data, code and other materials | 27 | Template data collection forms, data extracted from included studies, data used for all analyses are available. | P9 |

*From:*  Page MJ, McKenzie JE, Bossuyt PM, Boutron I, Hoffmann TC, Mulrow CD, et al. The PRISMA 2020 statement: an updated guideline for reporting systematic reviews. BMJ 2021;372:n71. doi: 10.1136/bmj.n71
