# Supplementary material for: The effect of periodontal treatments on endothelial function in degrees of periodontitis patients: A systematic review and meta-analysis
Source: PLoS One. 2024 Sep 19;19(9):e0308793. doi: 10.1371/journal.pone.0308793 (PMC11412498; doi:10.1371/journal.pone.0308793)
Supplement: S2 Table — (DOCX) [file pone.0308793.s002.docx]

**Supplemental Table 2 Search Strategy**

Search date:November,2023

| Database | Search term | results |
| --- | --- | --- |
| PubMed(175)  (Filters: Clinical Trial, Randomized Controlled Trial) | (endothelial function) AND (periodontal disease) Filters: Clinical Trial, Randomized Controlled Trial | 41 |
|  | (("Periodontitis/therapy"[Mesh])) AND (( "Endothelium, Vascular/abnormalities"[Mesh] OR "Endothelium, Vascular/diagnostic imaging"[Mesh] OR "Endothelium, Vascular/physiology"[Mesh] OR "Endothelium, Vascular/physiopathology"[Mesh] OR "Endothelium, Vascular/radiation effects"[Mesh] ) OR ("flow-mediated dilatation")) | 8 |
|  | ((((periodontitis) OR ("periodontitis therapy")) OR ("periodontitis treatment")) OR ("periodontitis nursing")) AND (((((("endothelial dysfunction") OR ("endothelial function")) OR ("flow-mediated-dilation")) OR ("endothelial vascular")) OR (cardiovascular)) OR (hypertension)) | 126 |

**Cochrane Library**

|  | Search term | results |
| --- | --- | --- |
| #1 | MeSH descriptor: [Subgingival Curettage] explode all trees | 1018 |
| #2 | MeSH descriptor: [Endothelium, Vascular] this term only | 3183 |
| #3 | MeSH descriptor: [Cardiovascular Disease] explode all trees and with quealifier(s): [pathology - PA] | 4142 |
| #4 | MeSH descriptor: [Hypertension] explode all trees and with qualifier(s): [complications - CO, pathology - PA] | 3647 |
| #5 | MeSH descriptor: [Root Planing] this term only | 919 |
| #6 | MeSH descriptor: [Dental Scaling] this term only | 1411 |
| #7 | MeSH descriptor: [Periodontics] explode all trees | 4283 |
| #8 | (flow-mediated dilatation): ti, ab, kw | 1254 |
| #9 | MeSH descriptor: [Periodontitis] explode all trees | 3975 |
| #10 | (#7 OR #6 OR #5 OR #1 OR #9) AND (#2 OR #3 OR #4 O #8) | 19 |
| #11 | periodontitis | 7520 |
| #12 | Endothelial function | 10293 |
| #13 | (#11) AND (#12) Filters: Trials | 46 |

**Web Of Science**

Query #1 (((TS=(periodontitis)) OR TS=(periodontitis therapy)) OR TS=(periodontitis treatment)) OR TS=(periodontitis nursing)

Query #2 (((((TS=(endothelial dysfunction)) OR TS=(endothelial function)) OR TS=(flow-mediated-dilation)) OR TS=(endothelial vascular)) OR TS=(cardiovascular)) OR TS=(hypertension)

Search: (#6) AND (#7) Filters: Clinical trial 24

Search: (periodontitis) AND TS=(endothelial function) and Article 319

**CNKI**

#1 “牙周炎” 和 “内皮功能” 41

#2 “牙周治疗” 和 “内皮功能” 16
