# Supplementary material for: The effect of periodontal treatments on endothelial function in degrees of periodontitis patients: A systematic review and meta-analysis
Source: PLoS One. 2024 Sep 19;19(9):e0308793. doi: 10.1371/journal.pone.0308793 (PMC11412498; doi:10.1371/journal.pone.0308793)
Supplement: S3 Table — (PDF) [file pone.0308793.s003.pdf]

Citation	Study location	Funding source	Study type	Sample size (M/F)	Age (years)	Systemic disease	Interventions	Follow-up time	Probing depth(mm) M±SD or M(IQR)	Clinical Attachment Loss(mm) M±SD or M(IQR)	BOP(%) M±SD or M(IQR)	Smoking Status no.(%)
Saffi MAL, 2018	South America（Brazil）	Brazilian Ministry of Science and Technology and the Research Support Agency from Rio Grande do Sul State	Randomized controlled trial	31(24/7)	58.6±8.5	Coronary artery disease	Supragingival and subgingival plaque removal、SRP	3 months	3.22 ± 0.54	5.12 ± 1.46	92.49 ± 10.10	No smoking before 6 months of treatment
Tonetti MS, 2007	England (London)	Grants from the University College London Hospital Research, Development Directorate, the British Heart Foundation, the European Research Group on Periodontology, the Periodontology Research Fund of the Eastman Dental Insti tute, Johnson & Johnson, the Coronary Artery Disease Research Association, the British Heart Foundation, the European Social Fund and	Randomized controlled trial	61(30/31)	47.7±7.9	NO	1. Full-mouth intensive removal of subgingival dental plaque biofilms with the use of scaling and root planing<br>2. Teeth that could not be saved were extracted<br>3. Microspheres of minocycline were delivered locally into the periodontal pockets	6 months	NM	NM	NM	Never smoked 24(39)<br>Former smoked 19(31)<br>Currrent smoker 18(31)

		Il Circolo, the Italian Society of Periodontology										
Ayako Okada, 2021	Asia（Japan）	Japan Society for the Promotion of Science (JSPS)	Open-label, randomized controlled trial	54（36/18)	32~45	NO	Full-mouth supragingival and subgingival plaque removal、SRP	3 months	2.1(1.9-2.4)	NM	10.8(5.9-20.0)	Current smoker 13(24)
Marcelo G. Lobo, 2020	South America（Brazil）	Instituto de Carrdiologia do Rio Grande do Sul	Randomized clinical trial	24(16/8)	52.7 ± 9.3	ST-segment elevation myocardial infarction	Supragingival and subgingival plaque removal、SRP	6 months	≥4mm 75%	≥4mm 90%	79%	NM
Biagio Rapone, 2022	Europe（Albania）	This research received no external funding	A Single-Blinded Randomized Clinical Trial	70（NM）	NM	NO	Whole-mouth supragingival and subgingival scaling and root planing of the teeth under local analgesia within 24 h.	3 months &6 months	5.73±0.5	5.87±0.48	NM	No smoking
Jorge Hernán Ramírez, 2014	South America（Columbia）	Universidad del Valle, Colombia	Randomized controlled trial	47(23/24)	⩾25	NO	One-stage full-mouth disinfection: scaling and root planing	3 months	NM	NM	NM	NM
John R. Elter DMD,2005	North Carolina	National Institutes of Health	Single-masked pilot clinical trial	22(12/10)	42	NO	Scaling and root planing	1 month	3.2±0.7	3.2±1.0	NM	Current smoker 1(5)
Seinost, G 2005	Austria	College	Clinical Investigation	30(11/19)	41.2	NO	1. Oral hygiene instruction, supragingival cleaning, scaling and root planning; 2. Systemic antimicrobial therapy was administered for 7 days and consisited of combination of amoxicillin plus clavulanic acid and metronidazole as adjunctive therapy	3 months	NM	NM	NM	Current smoker 8(26)
Fehmi Mercanoglu, 2004	Istanbul	College	Randomized clinical trial	28(21/7)	45.5 ± 8.6	NO	Full-Mouth Disinfection: Scaling and root planing	6 weeks	3.97±0.46	5.33±0.82	NM	NM

Arnon Blum, 2007	Israel	NM	Randomized clinical trial	22(10/12)	40±5	NO	1. An advanced oral hygiene improvement session followed by 2-4 consecutive sessions of scaling and root planning by one operatior;<br>2. Patients treated with antibiotics (amoxicillin 500 mg + metrinidazole 250 mg T.I.D.) during the first week.<br>Scaling and root planing	3 months	4.3	NM	64%	No smoking
XU JIA, 2017	China	NM	Randomized clinical trial	NM	NM	NO	1. Scaling and root planing；<br>2. Put 2% minocycline hydrochloride in periodontal pockets;<br>3. Extract teeth that cannot be saved	3 months	3.71±0.6	4.02±0.81	NM	NM
ZHENG YAO, 2011	China	NM	Randomized clinical trial	20(8/12)	36~45	NO	Scaling and root planing	3 months	5.11±0.51	3.22±0.33	NM	NM
JIAO RONG HONG, 2010	China	NM	Randomized clinical trial	18(8/10)	36~55	NO	Scaling and root planing	3 months	5.77±0.61	4.22±0.33	NM	NM
WANG TAO, 2013	China	NM	Randomized clinical trial	20(10/10)	26~59	NO	1. 2-4 times full-mouth scaling and root planing;<br>2. Patients treated with antibiotics (amoxicillin 500 mg + metrinidazole 250 mg T.I.D.) during the first week.	3 months	3.98±0.57	4.6±0.61	65%	No smoking
