# Supplementary material for: The effect of periodontal treatments on endothelial function in degrees of periodontitis patients: A systematic review and meta-analysis
Source: PLoS One. 2024 Sep 19;19(9):e0308793. doi: 10.1371/journal.pone.0308793 (PMC11412498; doi:10.1371/journal.pone.0308793)
Supplement: S4 Table — (PDF) [file pone.0308793.s004.pdf]

Author(s):
Question: Periodontal treatments compared to contral for FMD level
Setting:
Bibliography:

Certainty assessment							№ of patients		Effect		Certainty	Importance
**№ of studies**	**Study design**	**Risk of bias**	**Inconsistency**	**Indirectness**	**Imprecision**	**Other considerations**	**Periodontal treatments**	**contral**	**Relative (95% CI)**	**Absolute (95% CI)**		
**Arnon Blum,2007 (follow-up: range 1 months to 3 months; assessed with: FMD)**												
22	randomised trials	not serious	not serious	not serious	not serious	none	22	22	-	MD **7 lower** (10.44 lower to 3.56 lower)	⊕⊕⊕⊕ High	CRITICAL
**Ayako Okada,2021 (follow-up: range 1 months to 3 months; assessed with: FMD)**												
54	randomised trials	very serious[a,b]	not serious	not serious	not serious	all plausible residual confounding would reduce the demonstrated effect	54	54	-	MD **0.3 higher** (0.69 lower to 1.29 higher)	⊕⊕⊕◯ Moderate	IMPORTANT
**Biagio Rapone,2022 (follow-up: range 1 months to 3 months; assessed with: FMD)**												
70	randomised trials	serious[c]	not serious	not serious	not serious	dose response gradient	70	70	-	MD **0.38 lower** (0.58 lower to 0.18 lower)	⊕⊕⊕⊕ High	CRITICAL
**Jia Xu,2017 (follow-up: range 1 months to 3 months; assessed with: FMD)**												
43	randomised trials	very serious[b,c]	not serious	not serious	not serious	all plausible residual confounding would reduce the demonstrated effect	43	43	-	MD **1.45 lower** (2.89 lower to 0.01 lower)	⊕⊕⊕◯ Moderate	IMPORTANT
**John R. Elter,2005 (follow-up: range 1 months to 3 months; assessed with: FMD)**												
22	randomised trials	not serious	not serious	not serious	not serious	none	22	22	-	MD **1.6 lower** (4.15 lower to 0.95 higher)	⊕⊕⊕⊕ High	CRITICAL
**Jorge Hernán Ramírez,2014 (follow-up: range 1 months to 3 months; assessed with: FMD)**												
41	randomised trials	not serious	not serious	not serious	not serious	none	41	41	-	MD **1 lower** (4.72 lower to 2.72 higher)	⊕⊕⊕⊕ High	CRITICAL
**Marcelo G. Lobo, 2020 (follow-up: range 1 months to 6 months; assessed with: FMD)**												
24	randomised trials	not serious	not serious	not serious	not serious	none	24	24	-	MD **3.1 lower** (5.95 lower to 0.25 lower)	⊕⊕⊕⊕ High	CRITICAL
**Mercanoglu F,2004 (follow-up: mean 3 months; assessed with: FMD)**												
28	randomised trials	serious[b,c]	not serious	not serious	not serious	none	28	28	-	MD **9.3 lower** (11.88 lower to 6.72 lower)	⊕⊕⊕◯ Moderate	IMPORTANT
**Ronghong Jiao,2010 (follow-up: range 1 months to 3 months; assessed with: FMD)**												
18	randomised trials	very serious[b,c]	not serious	not serious	not serious	none	18	18	-	MD **7.15 lower** (9.56 lower to 4.47 lower)	⊕⊕◯◯ Low	NOT IMPORTANT
**Saffi MAL,2018 (follow-up: range 1 months to 3 months; assessed with: FMD)**												
38	randomised trials	not serious	not serious	not serious	not serious	none	38	38	-	MD **1.49 lower** (4.01 lower to 1.03 higher)	⊕⊕⊕⊕ High	CRITICAL
**Tao Wang,2013 (follow-up: range 1 months to 3 months; assessed with: FMD)**												
20	randomised trials	very serious[b,c]	not serious	not serious	not serious	all plausible residual confounding would reduce the demonstrated effect	20	20	-	MD **6.71 lower** (9.07 lower to 4.35 lower)	⊕⊕⊕◯ Moderate	IMPORTANT
**Tonetti MS,2007 (follow-up: range 1 months to 6 months; assessed with: FMD)**												
61	randomised trials	not serious	not serious	not serious	not serious	none	61	61	-	MD **1.2 lower** (2.51 lower to 0.11 higher)	⊕⊕⊕⊕ High	CRITICAL
**Yao Zheng,2011 (follow-up: range 1 months to 3 months; assessed with: FMD)**												

20	randomised trials	serious[c]	not serious	not serious	not serious	all plausible residual confounding would reduce the demonstrated effect	20	20	-	MD **7.15 lower** (9.02 lower to 5.28 lower)	⨁⨁⨁⨁ High	CRITICAL
**Seinost G,2005 (follow-up: range 1 months to 3 months; assessed with: FMD)**												
30	randomised trials	very serious[b,c]	not serious	not serious	not serious	all plausible residual confounding would reduce the demonstrated effect	30	30	-	MD **3.7 lower** (6.28 lower to 1.12 lower)	⨁⨁⨁◯ Moderate	CRITICAL

CI: confidence interval; **MD:** mean difference

Explanations

a. Deviations from intended intervention
b. Measurement of the outcome
c. randomization process
