# Supplementary material for: The effect of periodontal treatments on endothelial function in degrees of periodontitis patients: A systematic review and meta-analysis
Source: PLoS One. 2024 Sep 19;19(9):e0308793. doi: 10.1371/journal.pone.0308793 (PMC11412498; doi:10.1371/journal.pone.0308793)
Supplement: S5 Table — (DOCX) [file pone.0308793.s005.docx]

| **Citation** | **Registration** |
| --- | --- |
| Ayako Okada, 2021 | This trial is registered in UMIN Clinical Trials Registry ([www.umin.ac.jp/ctr/](https://www.umin.ac.jp/ctr/);ID:UMIN000023395). |
| Biagio Rapone,2022 | The trial is registered with the ISRCTN registry, number ISRCTN13626790 |
| Marcelo G.Lobo,2020 | This study was registered on [www.clinicaltrials.gov](http://www.clinicaltrials.gov/) under the number [NCT02543502](http://clinicaltrials.gov/show/NCT02543502) |
| Saffi MAL,2018 | The trial protocol was registered on ClinicalTrials.gov (identifier NCT01609725) |

**S5 Table. Experimental registration status**
