# Supplementary material for: The effect of periodontal treatments on endothelial function in degrees of periodontitis patients: A systematic review and meta-analysis
Source: PLoS One. 2024 Sep 19;19(9):e0308793. doi: 10.1371/journal.pone.0308793 (PMC11412498; doi:10.1371/journal.pone.0308793)
Supplement: S6 Table — (PDF) [file pone.0308793.s006.pdf]

S6 Table.Periodontitis criteria

	Citation	Include Criteria	Criteria for judging the severity of periodontitis
1	Ayako,Okada，2021	● age range of 20–70 years<br>● having ≥20 functioning teeth<br>● diagnosis of chronic periodontitis with ≥2 sites with bleeding on probing (BOP) or a probing pocket depth (PPD) of ≥4 mm at ≥1 site	NM
2	Biagio Rapone，2022	● each patient included a detailed medical and clinical history<br>● a full periodontal examination, blood collection, and flow-mediated brachial artery dilatation.	The severity of the periodontitis was determined according to the *Consensus Report of World Workshop on the Classification of Periodontal and Peri-Implant Diseases and Conditions[1]*
3	Blum, A, 2007	● each patient had at least 18 teeth and 1 interproximal area of 5 mm or greater pocket depth.<br>● none of them smoked or had any of the conventional risk factors for atherosclerosis (hypertension, high cholesterol or triglyceride levels, family history of premature coronary artery or vascular disease, diabetes mellitus, any immunological or known chronic inflammatory condition, or malignancy)	NM
4	Fehmi Mercanoglu, 2004	chronic periodontitis	Diagnosis of chronic periodontitis was based on clinical attachment loss and radiographic bone loss[2]
5	Jorge H Ramírez, 2011	● Male or female<br>● 25 years of age or older<br>● Three or more periodontal pockets with a probing depth (PD) > 5 mm<br>● Have at least 16 natural teeth excluding third molars	NM

6	John R. Elter, 2006	● age >30 years<br>● ≥1 tooth present in at least 2 quadrants<br>● moderate to severe chronic periodontal disease (at least 4 sites with	Classification of periodontal diseases. [3]
7	Marcelo G. Lobo, 2020	clinical attachment loss higher or equal to 4 mm and subgingival probing depth higher or equal to 6 mm in at least 5 teeth, associated to gingival bleeding in at least 8 teeth	Case Definitions for Use in Population-Based Surveillance of Periodontitis[4]
8	Marco Aurélio L. Saffi, 2018	the presence of at least two nonadjacent teeth with probing depth (PD) ≥5 mm and attachment loss (AL) ≥6 mm. Furthermore, subjects were required to have at least 10 teeth.	The diagnosis of periodontitis was defined in accordance with the Centers for Disease Control and the American Academy of Periodontology (CDC-AAP) criteria (Eke, Page, Wei, Thornton-Evans, & Genco, 2012)[5]
9	Gerald Seinost,2005	at least 6 teeth with pocket depth >5 mm and loss of attachment of ≥3 mm in 3 aspects of each involved tooth	Prevalence, extent, severity and progression of periodontal disease
10	Maurizio S. Tonetti,2007	probing pocket depths of >6 mm and marginal alveolar bone loss of >30% with 50% or more of their teeth affected	NM

[1] Papapanou, P.N.; Sanz, M.; Buduneli, N.; Dietrich, T.; Feres, M.; Fine, D.H.; Flemmig, T.F.; Garcia, R.; Giannobile, W.V.; Graziani, F.; et al. Periodontitis: Consensus report of workgroup 2 of the 2017 World Workshop on the Classification of Periodontal and Peri-Implant Diseases and Conditions. J. Periodontol. 2018, 89, S173–S182.
[2] Armitage GC. Development of a classification system for periodontal disease and conditions. Ann periodontol 1999;4:1-7
[3] Ranney, R.R., 1993. Classification of periodontal diseases. Periodontology 2000 2, 13–25. https://doi.org/10.1111/j.1600-0757.1993.tb00216.x
[4] Case Definitions for Use in Population-Based Surveillance of Periodontitis - Page - 2007 - Journal of Periodontology - Wiley Online Library [Internet]. [cited 2024 Jul 2]. Available from: https://aap.onlinelibrary.wiley.com/doi/10.1902/jop.2007.060264
[5] L.J. Brown, H. Loe Prevalence, extent, severity and progression of periodontal disease Periodontol 2000, 2 (1993), pp. 57-71
