# Supplementary material for: The effect of periodontal treatments on endothelial function in degrees of periodontitis patients: A systematic review and meta-analysis
Source: PLoS One. 2024 Sep 19;19(9):e0308793. doi: 10.1371/journal.pone.0308793 (PMC11412498; doi:10.1371/journal.pone.0308793)

Fig 4.1 Forest plot for comparison of changes in FMD levels after periodontal treatment in all 14 studies included

Study or Subgroup	Baseline Mean	Baseline SD	Baseline Total	PT Mean	PT SD	PT Total	Weight	Mean Difference IV, Random, 95% CI
Arnon Blum,2007	4.12	3.96	22	11.12	7.22	22	5.8%	-7.00 [-10.44, -3.56]
Ayako Okada,2021	5.8	2.9	54	5.5	2.3	54	8.3%	0.30 [-0.69, 1.29]
Biagio Rapone,2022	9.62	0.62	70	10	0.56	70	8.7%	-0.38 [-0.58, -0.18]
Jia Xu,2017	8.02	3.13	43	9.47	3.65	43	8.0%	-1.45 [-2.89, -0.01]
John R. Elter,2005	8.6	4.7	22	10.2	3.9	22	6.8%	-1.60 [-4.15, 0.95]
Jorge Hernán Ramírez,2014	10.2	7.3	41	11.2	9.7	41	5.5%	-1.00 [-4.72, 2.72]
Marcelo G. Lobo, 2020	9	4.4	24	12.1	5.6	24	6.5%	-3.10 [-5.95, -0.25]
Mercanoglu F,2004	8.4	4	28	17.7	5.7	28	6.8%	-9.30 [-11.88, -6.72]
Ronghong Jiao,2010	5.11	2.92	18	12.26	4.33	18	7.0%	-7.15 [-9.56, -4.74]
Saffi MAL,2018	7.05	5.6	38	8.54	5.6	38	6.9%	-1.49 [-4.01, 1.03]
Seinost G,2005	6.1	4.4	30	9.8	5.7	30	6.8%	-3.70 [-6.28, -1.12]
Tao Wang,2013	5.11	3.16	20	11.82	4.36	20	7.1%	-6.71 [-9.07, -4.35]
Tonetti MS,2007	7.1	4.2	61	8.3	3.1	61	8.1%	-1.20 [-2.51, 0.11]
Yao Zheng,2011	6.11	1.88	20	13.26	3.83	20	7.6%	-7.15 [-9.02, -5.28]
**Total (95% CI)**			**491**			**491**	**100.0%**	**-3.50 [-4.96, -2.05]**

Heterogeneity: $Tau^2 = 6.37$; $Chi^2 = 177.86$, $df = 13$ ($P < 0.00001$); $I^2 = 93\%$
Test for overall effect: $Z = 4.71$ ($P < 0.00001$)

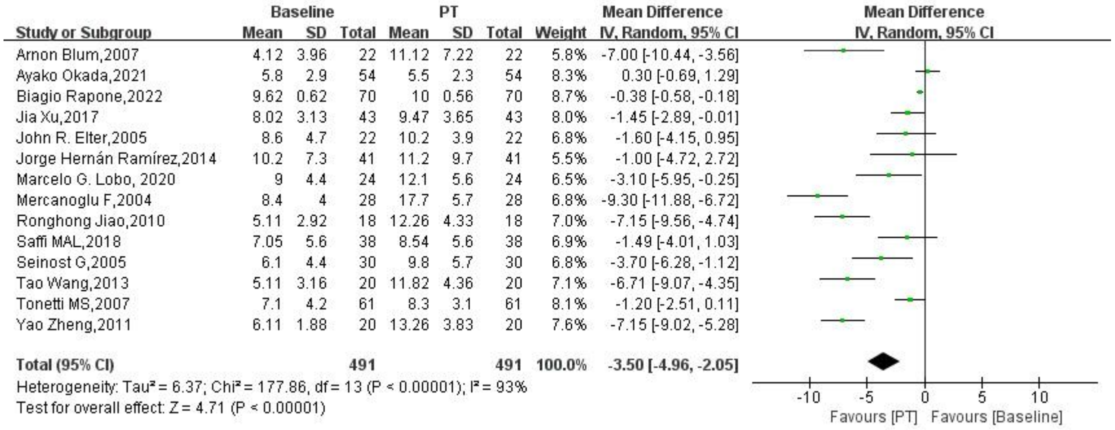

Fig 4.2 Funnel plot for comparison of changes in FMD levels after periodontal treatment in all 14 studies included

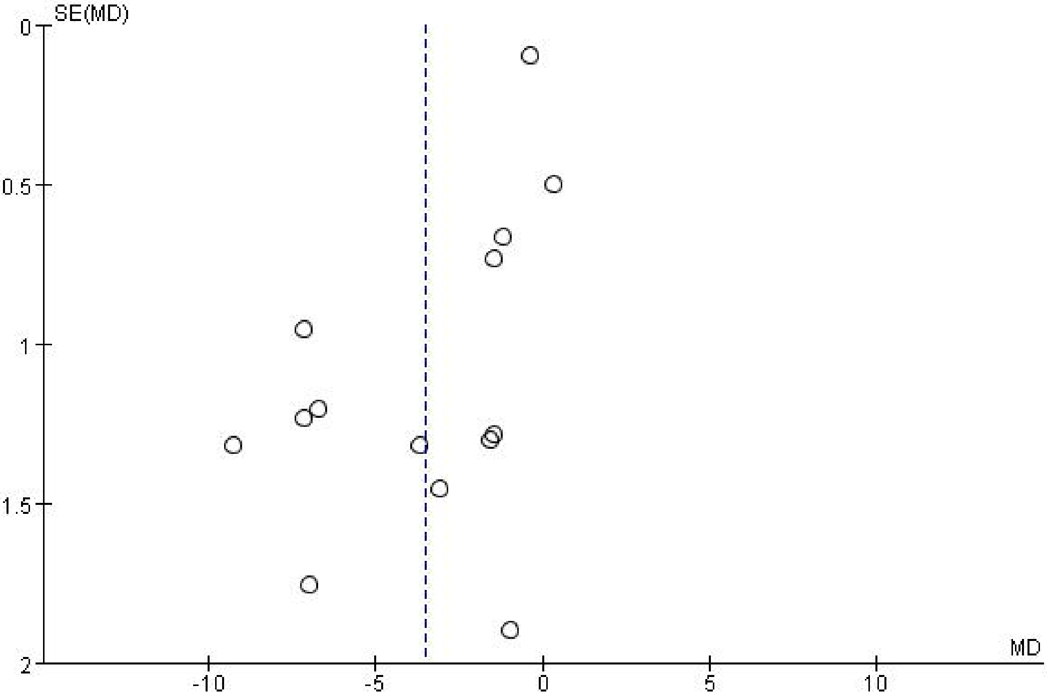

Fig 5.1 Forest plot for comparison of FMD levels after short-term (≤3 months) periodontal treatment

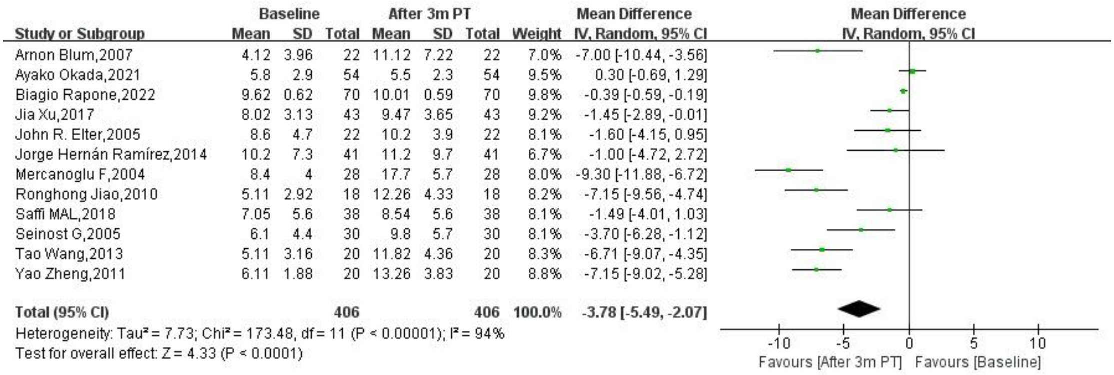

Study or Subgroup	Baseline Mean	Baseline SD	Baseline Total	After 3m PT Mean	After 3m PT SD	After 3m PT Total	Weight	Mean Difference IV, Random, 95% CI
Arnon Blum,2007	4.12	3.96	22	11.12	7.22	22	7.0%	-7.00 [-10.44, -3.56]
Ayako Okada,2021	5.8	2.9	54	5.5	2.3	54	9.5%	0.30 [-0.69, 1.29]
Biagio Rapone,2022	9.62	0.62	70	10.01	0.59	70	9.8%	-0.39 [-0.59, -0.19]
Jia Xu,2017	8.02	3.13	43	9.47	3.65	43	9.2%	-1.45 [-2.89, -0.01]
John R. Elter,2005	8.6	4.7	22	10.2	3.9	22	8.1%	-1.60 [-4.15, 0.95]
Jorge Hernán Ramírez,2014	10.2	7.3	41	11.2	9.7	41	6.7%	-1.00 [-4.72, 2.72]
Mercanoglu F,2004	8.4	4	28	17.7	5.7	28	8.0%	-9.30 [-11.88, -6.72]
Ronghong Jiao,2010	5.11	2.92	18	12.26	4.33	18	8.2%	-7.15 [-9.56, -4.74]
Saffi MAL,2018	7.05	5.6	38	8.54	5.6	38	8.1%	-1.49 [-4.01, 1.03]
Seinost G,2005	6.1	4.4	30	9.8	5.7	30	8.1%	-3.70 [-6.28, -1.12]
Tao Wang,2013	5.11	3.16	20	11.82	4.36	20	8.3%	-6.71 [-9.07, -4.35]
Yao Zheng,2011	6.11	1.88	20	13.26	3.83	20	8.8%	-7.15 [-9.02, -5.28]
**Total (95% CI)**			**406**			**406**	**100.0%**	**-3.78 [-5.49, -2.07]**

Heterogeneity: Tau² = 7.73; Chi² = 173.48, df = 11 (P < 0.00001); I² = 94%
Test for overall effect: Z = 4.33 (P < 0.0001)

Fig 5.2 Funnel plot for comparison of FMD levels after short-term (3 months) periodontal treatment

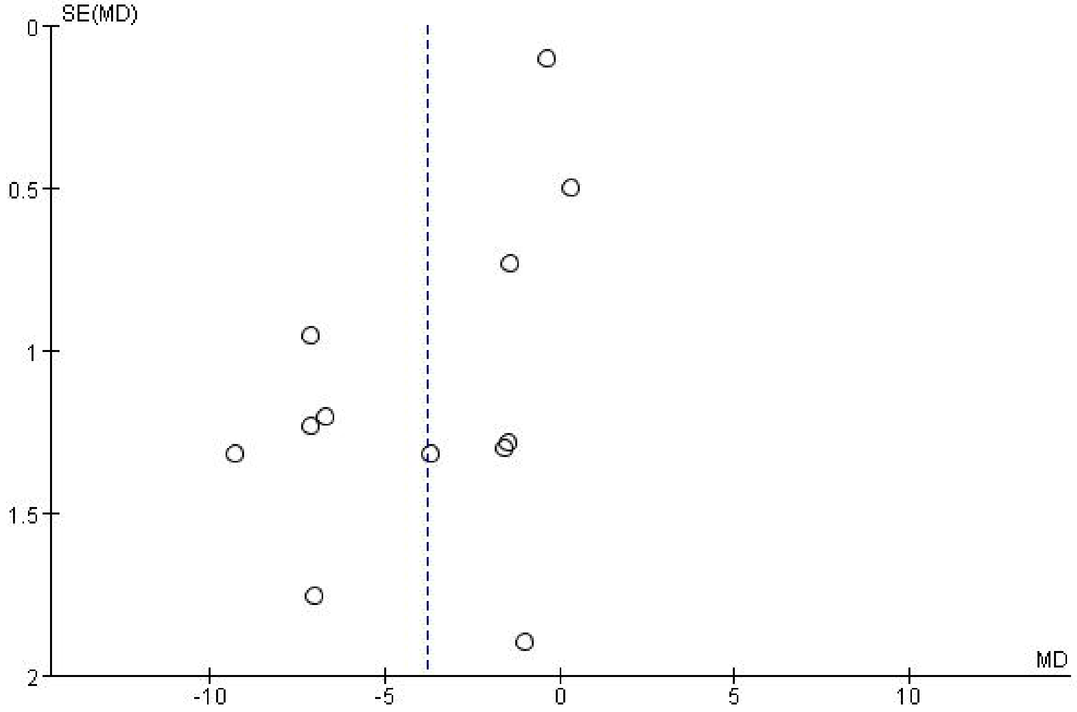

Fig 6.1 Forest plot for comparison of FMD levels after long-term (6 months) periodontal treatment

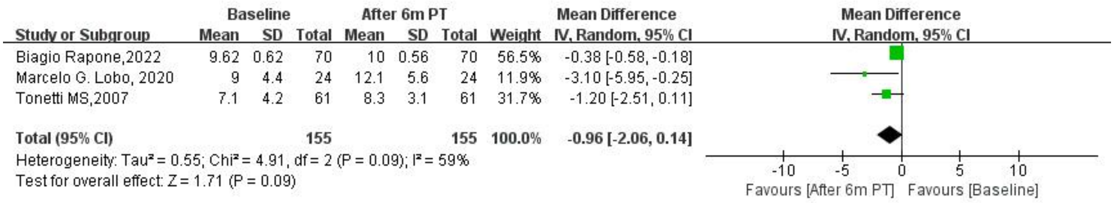

Fig 6.2 Funnel plot for comparison of FMD levels after long-term (6 months) periodontal treatment

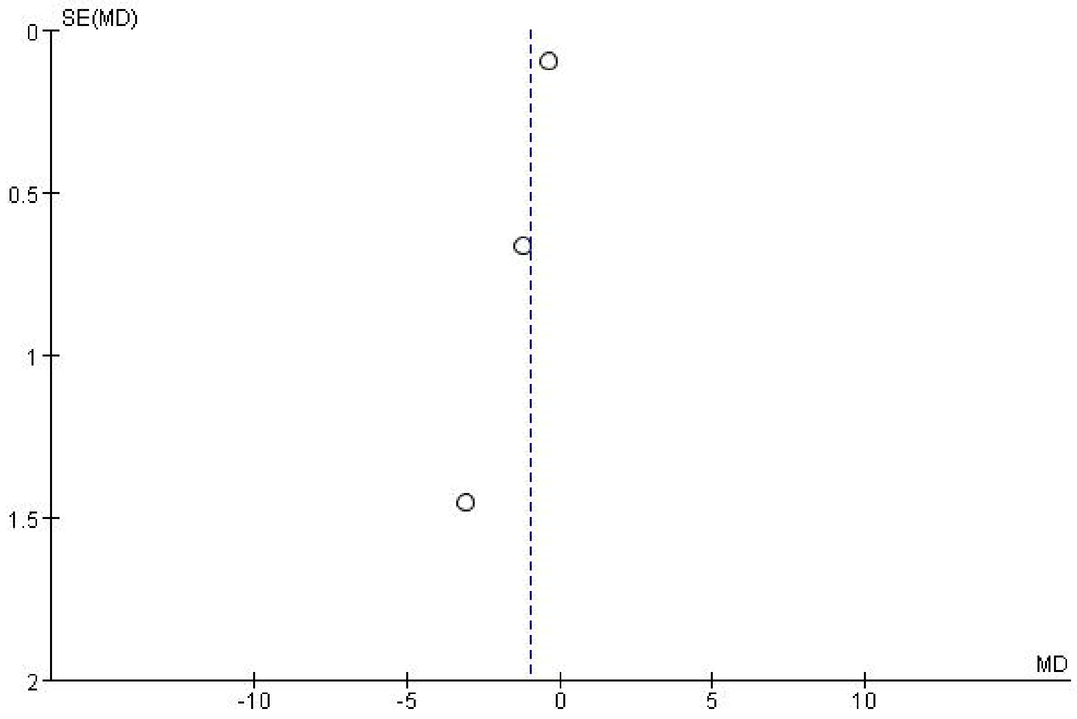

Fig 7.1 Forest plot for comparison of FMD levels after receiving periodontal treatment for severe periodontitis

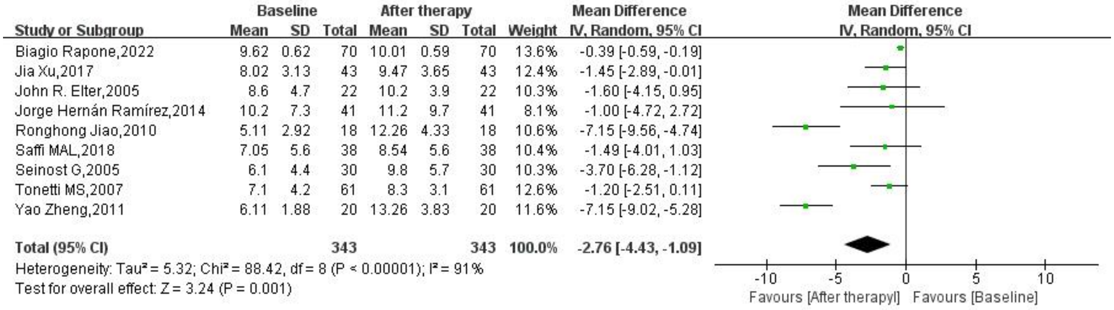

Fig 7.2 Funnel plot for comparison of FMD levels after receiving periodontal treatment for severe periodontitis

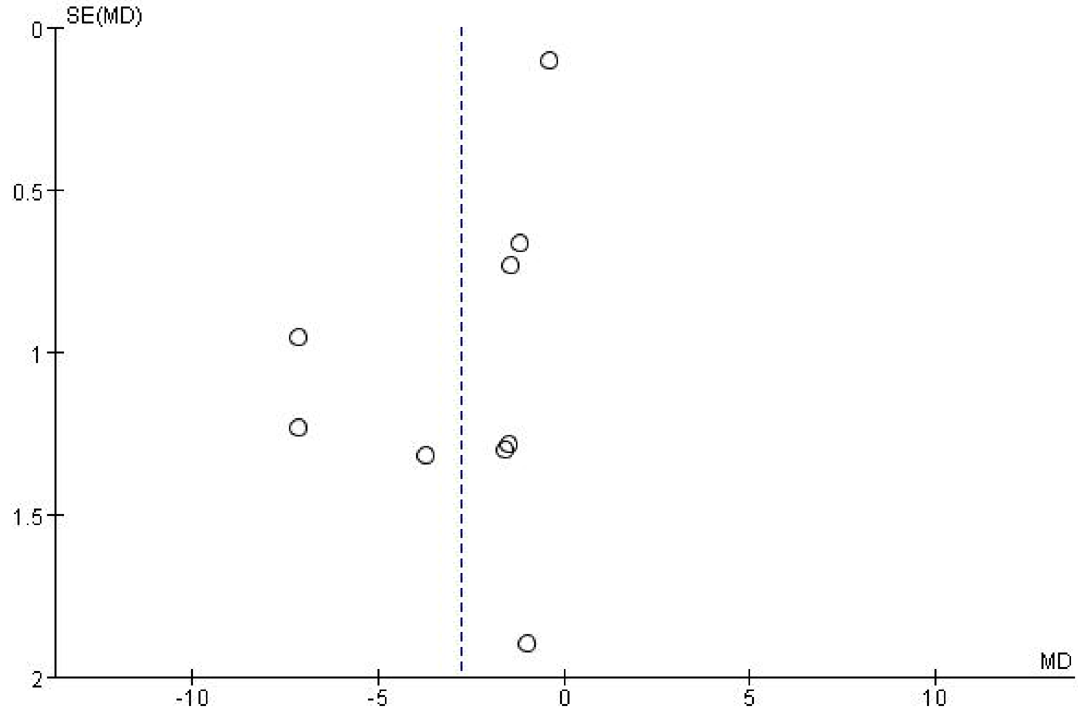

Fig 8.1 Forest plot for comparison of changes in FMD levels in periodontitis patients with cardiovascular disease after periodontal therapy

Study or Subgroup	Baseline Mean	Baseline SD	Baseline Total	After therapy Mean	After therapy SD	After therapy Total	Weight	Mean Difference IV, Random, 95% CI
Marcelo G. Lobo, 2020	9	4.4	24	12.1	5.6	24	43.9%	-3.10 [-5.95, -0.25]
Saffi MAL,2018	7.05	5.6	38	8.54	5.6	38	56.1%	-1.49 [-4.01, 1.03]
**Total (95% CI)**			**62**			**62**	**100.0%**	**-2.20 [-4.08, -0.31]**

Heterogeneity: Tau² = 0.00; Chi² = 0.69, df = 1 (P = 0.41); I² = 0%
Test for overall effect: Z = 2.28 (P = 0.02)

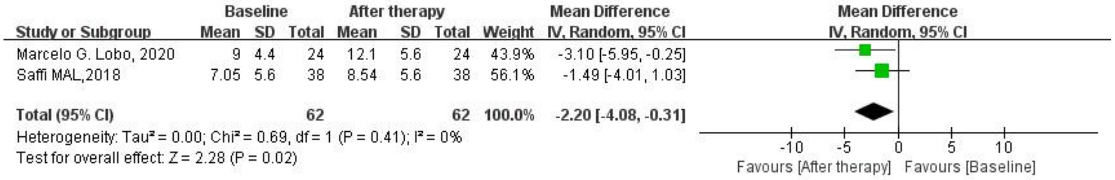

Fig 8.2 Funnel plot for comparison of changes in FMD levels in periodontitis patients with cardiovascular disease after periodontal therapy

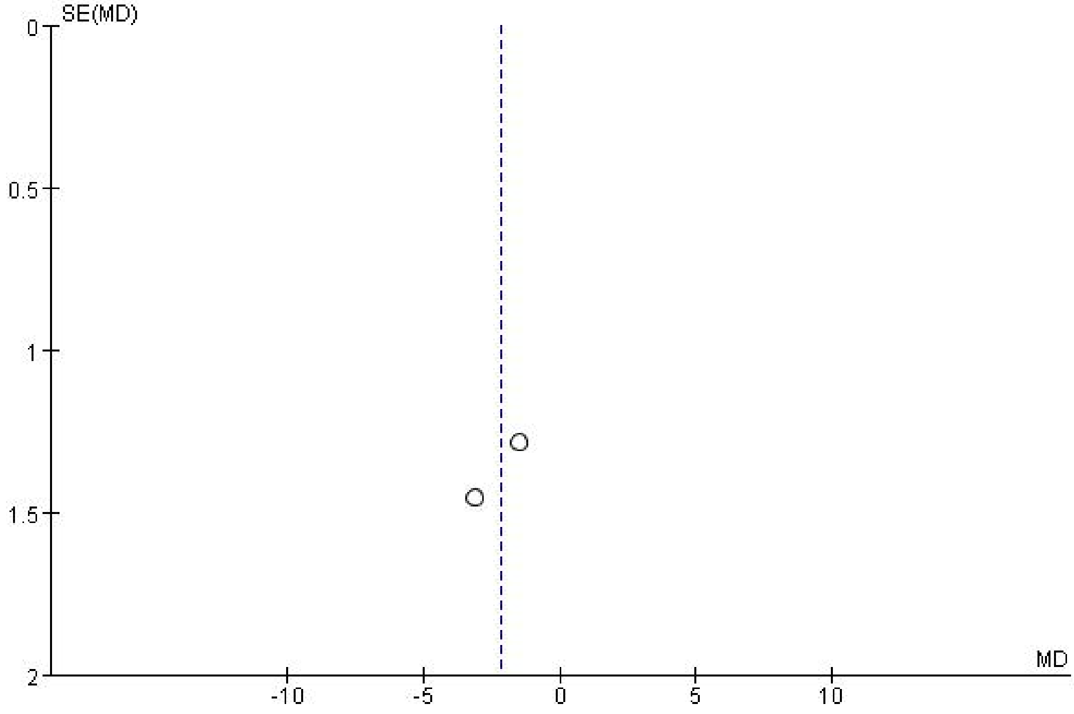

Fig 9.1 Forest plot for comparison of FMD level changes in patients with endothelial dysfunction after periodontal treatment

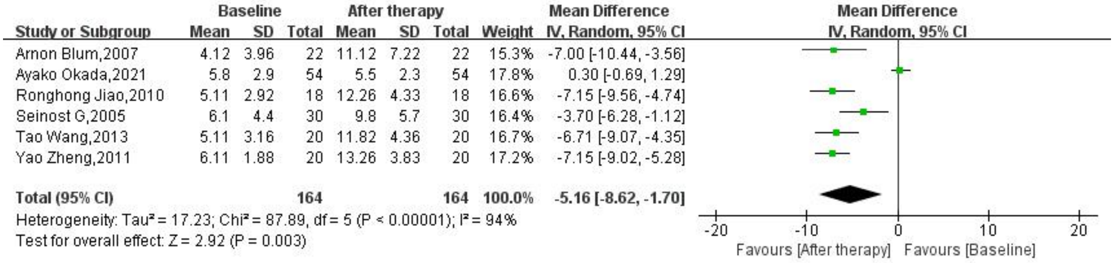

Study or Subgroup	Baseline Mean	Baseline SD	Baseline Total	After therapy Mean	After therapy SD	After therapy Total	Weight	Mean Difference IV, Random, 95% CI
Arnon Blum,2007	4.12	3.96	22	11.12	7.22	22	15.3%	-7.00 [-10.44, -3.56]
Ayako Okada,2021	5.8	2.9	54	5.5	2.3	54	17.8%	0.30 [-0.69, 1.29]
Ronghong Jiao,2010	5.11	2.92	18	12.26	4.33	18	16.6%	-7.15 [-9.56, -4.74]
Seinost G,2005	6.1	4.4	30	9.8	5.7	30	16.4%	-3.70 [-6.28, -1.12]
Tao Wang,2013	5.11	3.16	20	11.82	4.36	20	16.7%	-6.71 [-9.07, -4.35]
Yao Zheng,2011	6.11	1.88	20	13.26	3.83	20	17.2%	-7.15 [-9.02, -5.28]
**Total (95% CI)**			**164**			**164**	**100.0%**	**-5.16 [-8.62, -1.70]**

Heterogeneity: Tau² = 17.23; Chi² = 87.89, df = 5 (P < 0.00001); I² = 94%
Test for overall effect: Z = 2.92 (P = 0.003)

Fig 9.2 Funnel plot for comparison of FMD level changes in patients with endothelial dysfunction after periodontal treatment

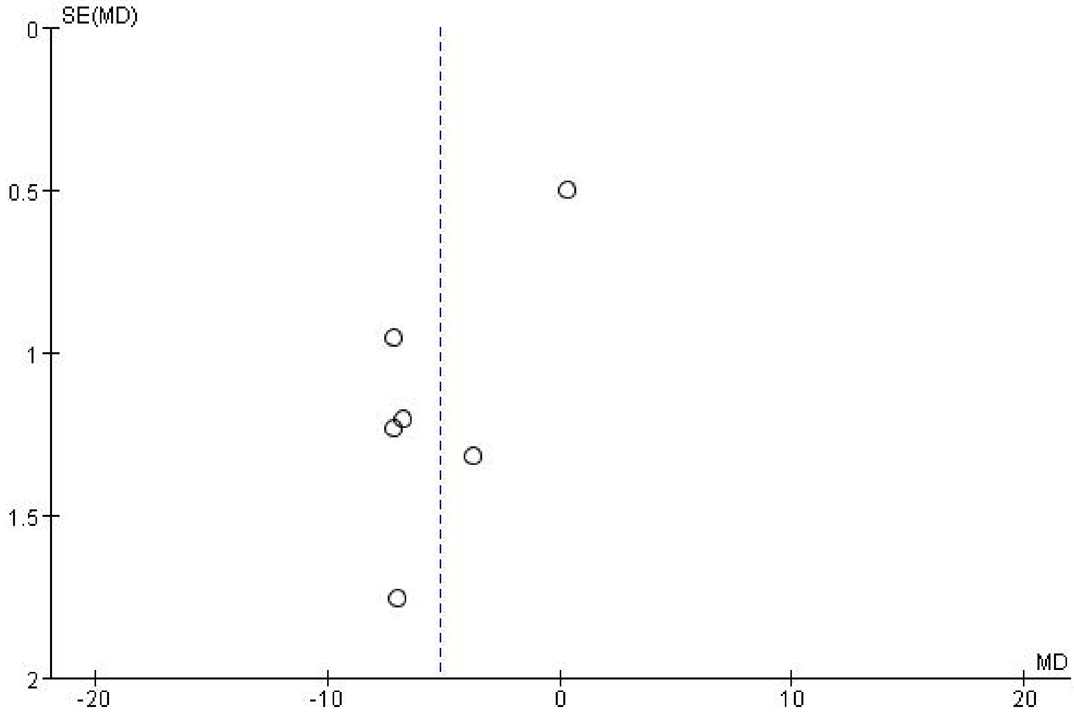

Fig 10.1 Forest plot for clinical trials that have been registered

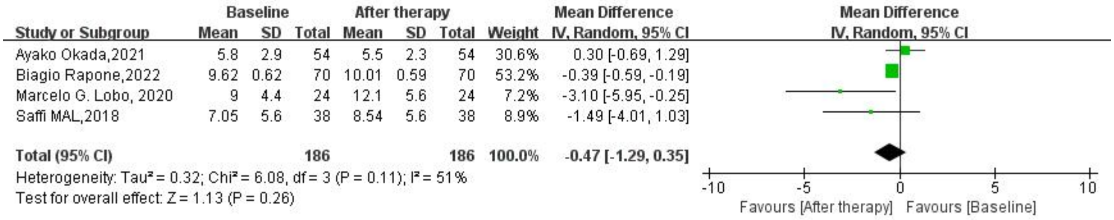

Fig 10.2 Funnel plot for clinical trials that have been registered

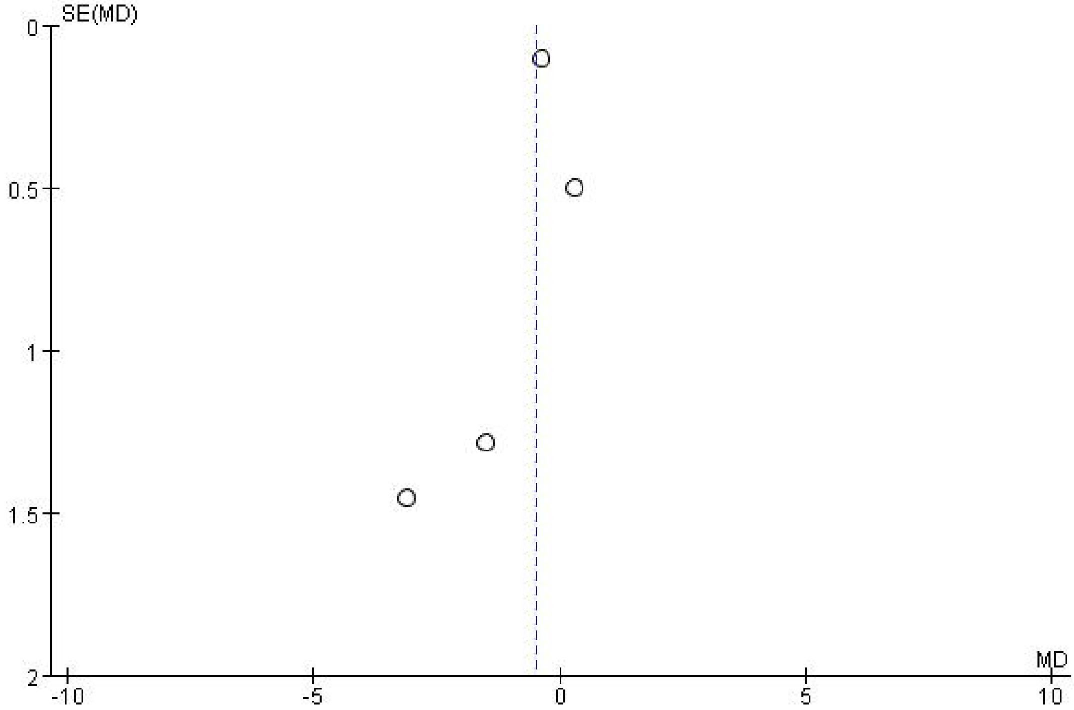

Fig 11.1 Forest plot for comparison of FMD levels after treatment with antimicrobial medication

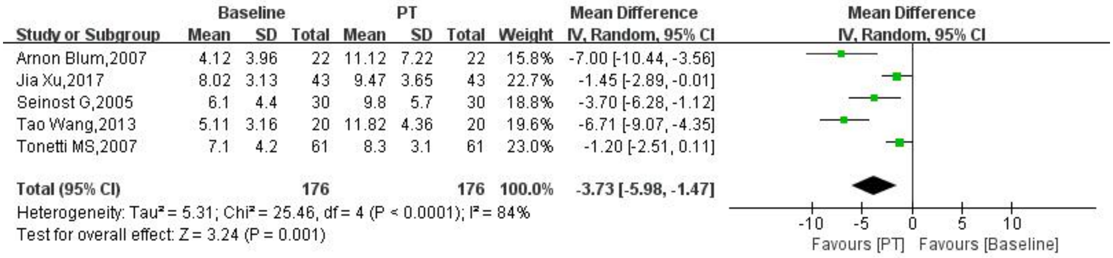

Study or Subgroup	Baseline Mean	Baseline SD	Baseline Total	PT Mean	PT SD	PT Total	Weight	Mean Difference IV, Random, 95% CI
Arnon Blum,2007	4.12	3.96	22	11.12	7.22	22	15.8%	-7.00 [-10.44, -3.56]
Jia Xu,2017	8.02	3.13	43	9.47	3.65	43	22.7%	-1.45 [-2.89, -0.01]
Seinost G,2005	6.1	4.4	30	9.8	5.7	30	18.8%	-3.70 [-6.28, -1.12]
Tao Wang,2013	5.11	3.16	20	11.82	4.36	20	19.6%	-6.71 [-9.07, -4.35]
Tonetti MS,2007	7.1	4.2	61	8.3	3.1	61	23.0%	-1.20 [-2.51, 0.11]
**Total (95% CI)**			**176**			**176**	**100.0%**	**-3.73 [-5.98, -1.47]**

Heterogeneity: Tau² = 5.31; Chi² = 25.46, df = 4 (P < 0.0001); I² = 84%
Test for overall effect: Z = 3.24 (P = 0.001)

Fig 11.2 Funnel plot for comparison of FMD levels after treatment with antimicrobial medication

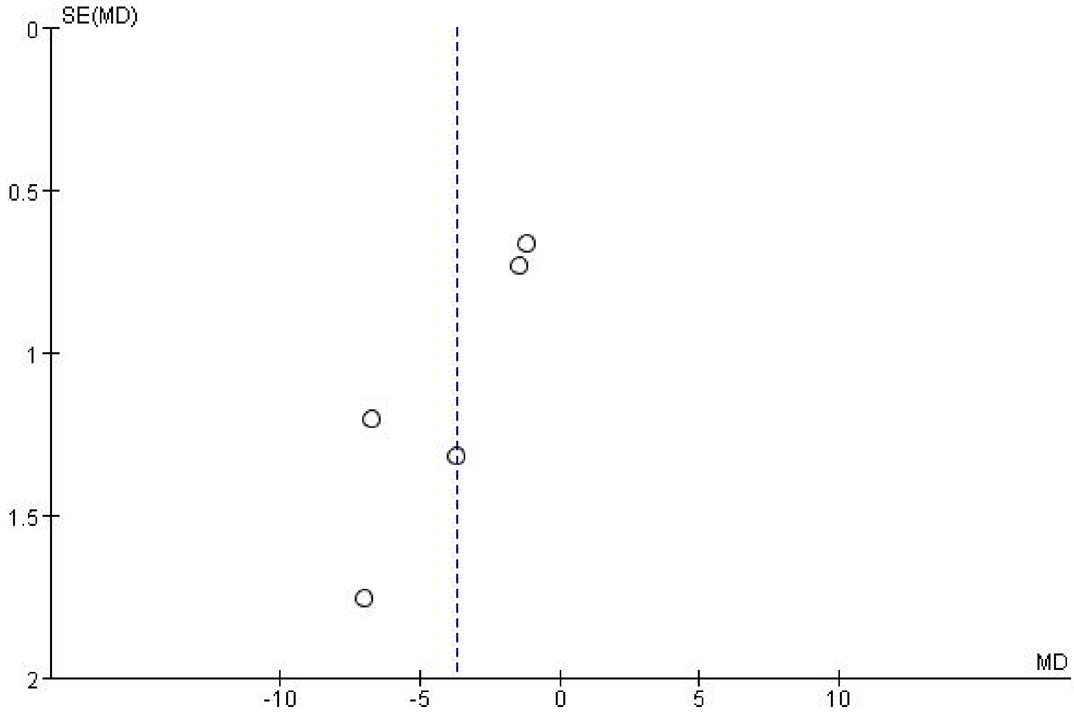

Fig 12.1 Forest plot for comparison of changes in FMD levels after extraction of affected teeth with no retention value

	Baseline			PT				Mean Difference
Study or Subgroup	Mean	SD	Total	Mean	SD	Total	Weight	IV, Random, 95% CI
Jia Xu,2017	8.02	3.13	43	9.47	3.65	43	45.4%	-1.45 [-2.89, -0.01]
Tonetti MS,2007	7.1	4.2	61	8.3	3.1	61	54.6%	-1.20 [-2.51, 0.11]
**Total (95% CI)**			**104**			**104**	**100.0%**	**-1.31 [-2.28, -0.35]**

Heterogeneity: Tau² = 0.00; Chi² = 0.06, df = 1 (P = 0.80); I² = 0%
Test for overall effect: Z = 2.66 (P = 0.008)

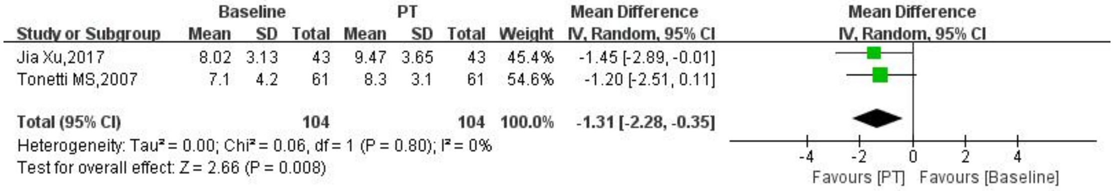

Fig 12.2 Funnel plot for comparison of changes in FMD levels after extraction of affected teeth with no retention value

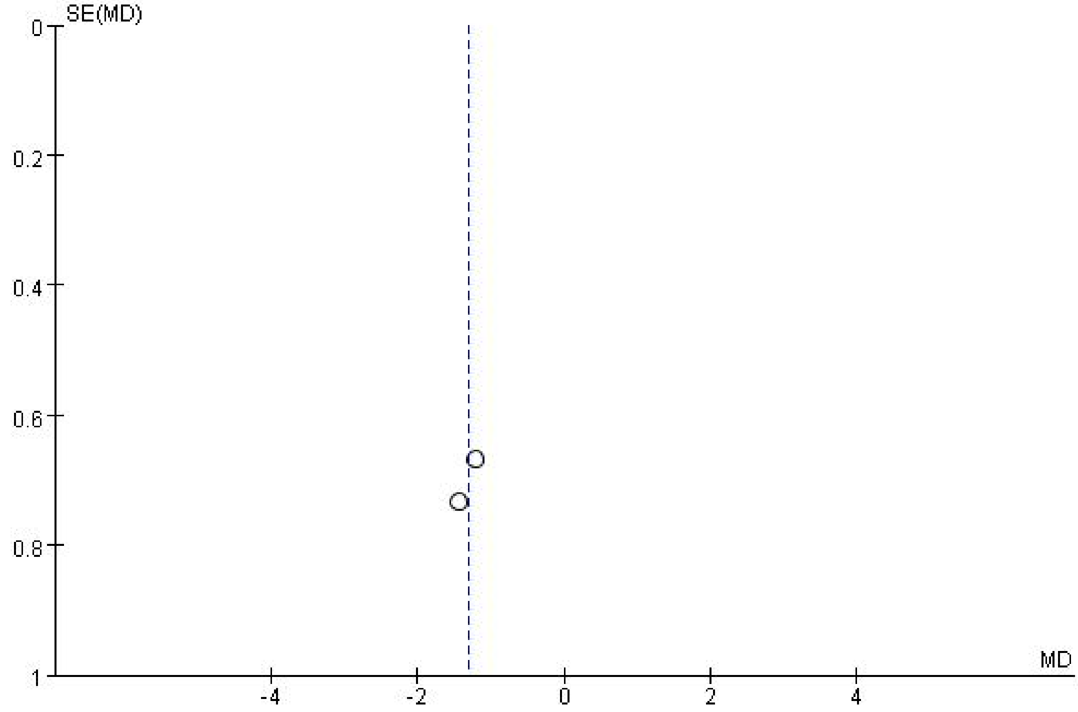

Supplement: S1 Appendix — (PDF) [file pone.0308793.s007.pdf]
