# Supplementary material for: The effect of periodontal treatments on endothelial function in degrees of periodontitis patients: A systematic review and meta-analysis
Source: PLoS One. 2024 Sep 19;19(9):e0308793. doi: 10.1371/journal.pone.0308793 (PMC11412498; doi:10.1371/journal.pone.0308793)
Supplement: S2 Appendix — (DOCX) [file pone.0308793.s008.docx]

**S9 Appendix. Supporting references**

1. Beck JD, Elter JR, Heiss G, Couper D, Mauriello SM, Offenbacher S. Relationship of periodontal disease to carotid artery intima-media wall thickness: the atherosclerosis risk in communities (ARIC) study. Arterioscler Thromb Vasc Biol. 2001 Nov;21(11):1816–22. PMID: 11701471
2. Mattila KJ, Valle MS, Nieminen MS, Valtonen VV, Hietaniemi KL. Dental infections and coronary atherosclerosis. Atherosclerosis. 1993; 103: 205–211. PMID: 8292096
3. Libby P, Ridker PM, Maseri A. Inflammation and atherosclerosis. Circulation. 2002 Mar 5;105(9):1135–43. PMID: 11877368
4. Hill CE, Phillips JK, Sandow SL. Heterogeneous control of blood flow amongst different vascular beds. Med Res Rev. 2001 Jan;21(1):1–60.PMID: 11135298
5. Widlansky ME, Gokce N, Keaney JF Jr, Vita JA. The clinical implications of endothelial dysfunction. J Am Coll Cardiol. 2003 Oct 1;42(7):1149–60. PMID: 14522472
6. Corretti MC, Anderson TJ, Benjamin EJ, Celermajer D, Charbonneau F, Creager MA, et al. Guidelines for the ultrasound assessment of endothelial-dependent flow-mediated vasodilation of the brachial artery: a report of the International Brachial Artery Reactivity Task Force. J Am Coll Cardiol. 2002 Jan 16;39(2):257–65. PMID: 11788217
7. Verma S, Buchanan MR, Anderson TJ. Endothelial function testing as a biomarker of vascular disease. Circulation. 2003 Oct 28;108(17):2054–9.PMID: 14581384
8. Alexander Y, Osto E, Schmidt-Trucksäss A, Shechter M, Trifunovic D, Duncker DJ, et al. Endothelial function in cardiovascular medicine: a consensus paper of the European Society of Cardiology Working Groups on Atherosclerosis and Vascular Biology, Aorta and Peripheral Vascular Diseases, Coronary Pathophysiology and Microcirculation, and Thrombosis. Cardiovasc Res. 2021 Jan 1;117(1):29–42.PMID: 32282914
9. Shabani Varaki E, Gargiulo GD, Penkala S, Breen PP. Peripheral vascular disease assessment in the lower limb: a review of current and emerging non-invasive diagnostic methods. Biomed Eng Online. 2018 May 11;17(1):61.PMID: 29751811
10. van Mil ACCM, Greyling A, Zock PL, Geleijnse JM, Hopman MT, Mensink RP, et al. Impact of volunteer-related and methodology-related factors on the reproducibility of brachial artery flow-mediated vasodilation: analysis of 672 individual repeated measurements. J Hypertens. 2016 Sep;34(9):1738–45.PMID: 27488550
11. Orlandi M, Suvan J, Petrie A, Donos N, Masi S, Hingorani A, et al. Association between periodontal disease and its treatment, flow-mediated dilatation and carotid intima-media thickness: a systematic review and meta-analysis. Atherosclerosis. 2014 Sep;236(1):39–46. PMID: 25014033
12. Teeuw WJ, Slot DE, Susanto H, Gerdes VEA, Abbas F, D’Aiuto F, et al. Treatment of periodontitis improves the atherosclerotic profile: a systematic review and meta-analysis. J Clin Periodontol. 2014 Jan;41(1):70–9.PMID: 24111886
13. Hada DS, Garg S, Ramteke GB, Ratre MS. Effect of Non-Surgical Periodontal Treatment on Clinical and Biochemical Risk Markers of Cardiovascular Disease: A Randomized Trial. J Periodontol. 2015 Nov;86(11):1201–11.PMID: 26205747
14. Offenbacher S, Beck JD, Moss K, Mendoza L, Paquette DW, Barrow DA, et al. Results from the Periodontitis and Vascular Events (PAVE) Study: a pilot multicentered, randomized, controlled trial to study effects of periodontal therapy in a secondary prevention model of cardiovascular disease. J Periodontol. 2009 Feb;80(2):190–201.PMID: 19186958
15. Vidal F, Figueredo CMS, Cordovil I, Fischer RG. Periodontal therapy reduces plasma levels of interleukin-6, C-reactive protein, and fibrinogen in patients with severe periodontitis and refractory arterial hypertension. J Periodontol. 2009 May;80(5):786–91.PMID: 19405832
16. Celermajer DS, Sorensen KE, Gooch VM, Spiegelhalter DJ, Miller OI, Sullivan ID, et al. Non-invasive detection of endothelial dysfunction in children and adults at risk of atherosclerosis. Lancet. 1992 Nov 7;340(8828):1111–5.PMID: 1359209
17. Deanfield JE, Halcox JP, Rabelink TJ. Endothelial function and dysfunction: testing and clinical relevance. Circulation. 2007 Mar 13;115(10):1285–95.PMID: 17353456
18. Morimoto H, Kajikawa M, Oda N, Idei N, Hirano H, Hida E, et al. Endothelial Function Assessed by Automatic Measurement of Enclosed Zone Flow‐Mediated Vasodilation Using an Oscillometric Method Is an Independent Predictor of Cardiovascular Events. JAHA. 2016 Dec 19;5(12).PMID: 28003249
19. Higgins JPT, Thompson SG. Quantifying heterogeneity in a meta-analysis. Stat Med. 2002 Jun 15;21(11):1539–58.PMID: 12111919
20. J. Li. Noninvasive evaluation of vascular endothelial function,from scientific research to clinic. Chinese Cardiovascular Journal,2019,24(05):411-413.
21. Amar S, Gokce N, Morgan S, Loukideli M, Van Dyke TE, Vita JA. Periodontal disease is associated with brachial artery endothelial dysfunction and systemic inflammation. Arterioscler Thromb Vasc Biol. 2003 Jul 1;23(7):1245–9.PMID: 12763762
22. Higashi Y, Goto C, Hidaka T, Soga J, Nakamura S, Fujii Y, et al. Oral infection-inflammatory pathway, periodontitis, is a risk factor for endothelial dysfunction in patients with coronary artery disease. Atherosclerosis. 2009 Oct;206(2):604–10.PMID: 19410250
23. D’Aiuto F, Parkar M, Tonetti MS. Acute effects of periodontal therapy on bio-markers of vascular health. J Clin Periodontol. 2007 Feb;34(2):124–9.PMID: 17214734
24. Pischon N, Hägewald S, Kunze M, Heng N, Christan C, Kleber BM, et al. Influence of periodontal therapy on the regulation of soluble cell adhesion molecule expression in aggressive periodontitis patients. J Periodontol. 2007 Apr;78(4):683–90.PMID: 17397316
25. Vidal F, Figueredo CMS, Cordovil I, Fischer RG. Periodontal therapy reduces plasma levels of interleukin-6, C-reactive protein, and fibrinogen in patients with severe periodontitis and refractory arterial hypertension. J Periodontol. 2009 May;80(5):786–91.PMID: 19405832
26. Parchure N, Zouridakis EG, Kaski JC. Effect of azithromycin treatment on endothelial function in patients with coronary artery disease and evidence of Chlamydia pneumoniae infection. Circulation. 2002 Mar 19;105(11):1298–303.PMID: 11901039
27. Graziani F, D’Aiuto F, Gennai S, Petrini M, Nisi M, Cirigliano N, et al. Systemic Inflammation after Third Molar Removal: A Case-Control Study. J Dent Res. 2017 Dec;96(13):1505–12.PMID: 28759304
