# Supplementary material for: The effect of periodontal treatments on endothelial function in degrees of periodontitis patients: A systematic review and meta-analysis
Source: PLoS One. 2024 Sep 19;19(9):e0308793. doi: 10.1371/journal.pone.0308793 (PMC11412498; doi:10.1371/journal.pone.0308793)
Supplement: S1 Dataset — (PDF) [file pone.0308793.s009.pdf]

Data and analyses

1 Data 1

1.1 All data from the 14 papers included

Study ID	Baseline			PT		
	Mean	SD	Total	Mean	SD	Total
Arnon Blum,2007	4.12	3.96	22	11.12	7.22	22
Ayako Okada,2021	5.8	2.9	54	5.5	2.3	54
Biagio Rapone,2022	9.62	0.62	70	10	0.56	70
Jia Xu,2017	8.02	3.13	43	9.47	3.65	43
John R. Elter,2005	8.6	4.7	22	10.2	3.9	22
Jorge Hernán Ramírez,2014	10.2	7.3	41	11.2	9.7	41
Marcelo G. Lobo, 2020	9	4.4	24	12.1	5.6	24
Mercanoglu F,2004	8.4	4	28	17.7	5.7	28
Ronghong Jiao,2010	5.11	2.92	18	12.26	4.33	18
Saffi MAL,2018	7.05	5.6	38	8.54	5.6	38
Seinost G,2005	6.1	4.4	30	9.8	5.7	30
Tao Wang,2013	5.11	3.16	20	11.82	4.36	20
Tonetti MS,2007	7.1	4.2	61	8.3	3.1	61
Yao Zheng,2011	6.11	1.88	20	13.26	3.83	20

Data and analyses

2 Data 2

2.1 Data on FMD levels after 3 months of periodontal treatment

Study ID	Baseline			After 3m PT		
	Mean	SD	Total	Mean	SD	Total
Arnon Blum,2007	4.12	3.96	22	11.12	7.22	22
Ayako Okada,2021	5.8	2.9	54	5.5	2.3	54
Biagio Rapone,2022	9.62	0.62	70	10.01	0.59	70
Jia Xu,2017	8.02	3.13	43	9.47	3.65	43
John R. Elter,2005	8.6	4.7	22	10.2	3.9	22
Jorge Hernán Ramírez,2014	10.2	7.3	41	11.2	9.7	41
Mercanoglu F,2004	8.4	4	28	17.7	5.7	28
Ronghong Jiao,2010	5.11	2.92	18	12.26	4.33	18
Saffi MAL,2018	7.05	5.6	38	8.54	5.6	38
Seinost G,2005	6.1	4.4	30	9.8	5.7	30
Tao Wang,2013	5.11	3.16	20	11.82	4.36	20
Yao Zheng,2011	6.11	1.88	20	13.26	3.83	20

Data and analyses

3 Data 3

3.1 Data on FMD levels after 6 months of periodontal treatment

Study ID	Baseline			After 6m PT		
	Mean	SD	Total	Mean	SD	Total
Biagio Rapone,2022	9.62	0.62	70	10	0.56	70
Marcelo G. Lobo, 2020	9	4.4	24	12.1	5.6	24
Tonetti MS,2007	7.1	4.2	61	8.3	3.1	61

Data and analyses

4 Data 4

4.1 Data on patients with severe periodontitis

Study ID	Baseline			After therapy		
	Mean	SD	Total	Mean	SD	Total
Biagio Rapone,2022	9.62	0.62	70	10.01	0.59	70
Jia Xu,2017	8.02	3.13	43	9.47	3.65	43
John R. Elter,2005	8.6	4.7	22	10.2	3.9	22
Jorge Hernán Ramírez,2014	10.2	7.3	41	11.2	9.7	41
Ronghong Jiao,2010	5.11	2.92	18	12.26	4.33	18
Saffi MAL,2018	7.05	5.6	38	8.54	5.6	38
Seinost G,2005	6.1	4.4	30	9.8	5.7	30
Tonetti MS,2007	7.1	4.2	61	8.3	3.1	61
Yao Zheng,2011	6.11	1.88	20	13.26	3.83	20

Data and analyses

5 Data 5

5.1 Data on patients with cardiovascular diseases

Study ID	Baseline			After therapy		
	Mean	SD	Total	Mean	SD	Total
Marcelo G. Lobo, 2020	9	4.4	24	12.1	5.6	24
Saffi MAL,2018	7.05	5.6	38	8.54	5.6	38

Data and analyses

6 Data 6

6.1 Data on patients with endothelial dysfunction

Study ID	Baseline			After therapy		
	Mean	SD	Total	Mean	SD	Total
Arnon Blum,2007	4.12	3.96	22	11.12	7.22	22
Ayako Okada,2021	5.8	2.9	54	5.5	2.3	54
Ronghong Jiao,2010	5.11	2.92	18	12.26	4.33	18
Seinost G,2005	6.1	4.4	30	9.8	5.7	30
Tao Wang,2013	5.11	3.16	20	11.82	4.36	20
Yao Zheng,2011	6.11	1.88	20	13.26	3.83	20

Data and analyses

7 Data 7

7.1 Data on clinical trials that have been registrered

Study ID	Baseline			After therapy		
	Mean	SD	Total	Mean	SD	Total
Ayako Okada,2021	5.8	2.9	54	5.5	2.3	54
Biagio Rapone,2022	9.62	0.62	70	10.01	0.59	70
Marcelo G. Lobo, 2020	9	4.4	24	12.1	5.6	24
Saffi MAL,2018	7.05	5.6	38	8.54	5.6	38

Data and analyses

8 Data 8

8.1 Data on the addition of antimicrobial drug to treatment

Study ID	Baseline			PT		
	Mean	SD	Total	Mean	SD	Total
Arnon Blum,2007	4.12	3.96	22	11.12	7.22	22
Jia Xu,2017	8.02	3.13	43	9.47	3.65	43
Seinost G,2005	6.1	4.4	30	9.8	5.7	30
Tao Wang,2013	5.11	3.16	20	11.82	4.36	20
Tonetti MS,2007	7.1	4.2	61	8.3	3.1	61

Data and analyses

9 Data 9

9.1 Data on extracting teeth in treatment

Study ID	Baseline			PT		
	Mean	SD	Total	Mean	SD	Total
Jia Xu,2017	8.02	3.13	43	9.47	3.65	43
Tonetti MS,2007	7.1	4.2	61	8.3	3.1	61
